# d-Amino Acid Position Influences the Anticancer Activity of Galaxamide Analogs: An Apoptotic Mechanism Study

**DOI:** 10.3390/ijms18030544

**Published:** 2017-03-10

**Authors:** Defa Bai, Siming Yu, Shenghui Zhong, Bingxin Zhao, Shaoling Qiu, Jianwei Chen, Jignesh Lunagariya, Xiaojian Liao, Shihai Xu

**Affiliations:** 1 College of Pharmacy, Jinan University, Guangzhou 510632, China; baidf@foxmail.com; 2 Key Laboratory of Biomaterials of Guangdong Higher Education Institutes, Department of Biomedical Engineering, Jinan University, Guangzhou 510632, China; siming_yu@hotmail.com; 3 Department of Chemistry, College of Chemistry and Materials Science, Jinan University, Guangzhou 510632, China; zhongsh@stu2014.jnu.edu.cn (S.Z.); zbx840622@163.com (B.Z.); qslhappyrain@gmail.com (S.Q.); 18826237580@163.com (J.C.); jignesh.lunagariya@gmail.com (J.L.)

**Keywords:** Galaxamide analogs, cyclopeptide, d-amino acid, anticancer, mechanism

## Abstract

Galaxamide, an extract from *Galaxaura filamentosa*, is a cyclic pentapeptide containing five l-leucines. Due to the particular cyclic structure and the excellent anticancer activity, synthesis of Galaxamide and its analogs and their subsequent bio-applications have attracted great attention. In the present work, we synthesized six Galaxamide analogs by replacing one of the l-leucines with phenylalanine and varying the d-amino acid position. The anticancer effect of the synthesized Galaxamide analogs was tested against four in vitro human cancer cell lines, human hepatocellular cells (HepG_2_), human breast cancer cell (MCF-7), human breast adenocarcinoma cells (MDA-MB-435) and a human cervical carcinoma cell line (Hela). Results showed that Galaxamide analogs with different d-amino acid positions displayed distinct anticancer potential. The Galaxamide analog containing d-amino acid at position 5 (Analog-6) presented the strongest anticancer activity. The mechanism study revealed that Analog-6 could cause the early apoptosis of HepG_2_ cells by inhibiting their growth in the sub-G1 stage of the cell cycle and induce the chromatin condensation and fragmentation, which can be seen as 68% of HepG_2_ cells inhibited in the sub-G1 stage. Moreover, a mitochondria-mediated pathway was found to be involved in the apoptotic process of Analog-6 on HepG_2_ cells.

## 1. Introduction

Cyclopeptides are a kind of peptide with a cyclic structure, which are widely distributed in nature. Because of the particular cyclic structure, cyclopeptides present many advantages over their linear counterparts, including: (1) cyclopeptides have a slower degradation behavior than the linear ones due to the fact that proteases can hardly access the cycle hole and cleave the amide bonds [1]; (2) the cyclopeptides bind more strongly for protein targets thanks to their constrained bond rotation and conformation [2]. Moreover, many recent studies have demonstrated the potential use of cyclopeptides as a clinical drug, such as antitumor, antiviral anti-inflammatory and antibiotic [3,4]. Therefore, the study of cyclopeptides has attracted enormous attention and becomes one of the most important parts of the research and development of antitumor drugs.

Cyclopeptides are mainly extracted from marine species. Since the first bioactive cyclopeptide ulithiacyclamide was isolated from the sea squirt by scientist of Chris Ireland in 1980 [5], more and more cyclopeptides have been isolated from various marine animals during the past three decades. Those cyclopeptides have been used as important resources in designing and developing novel antitumor drugs. Among those, cyclic-pentapeptide became the focus of our research group.

In our initial work, a novel cyclic-pentapeptide named Galaxamide was isolated from a marine algae *Galaxaura filamentosa* collected from Xisha Island in the South China Sea [6]. Galaxamide is a cyclic-pentapeptide composed of five *L-*leucines, including two *N*-methylated ones. *N*-methylated amino acid is an important component endowing the anticancer potential to the cyclopeptides [7]. We found that Galaxamide exhibited strong in vitro antiproliferative activities against human renal cell carcinoma GRC-1 and human hepatocellular carcinoma HepG_2_ cell lines, remarkably with half maximal inhibitory concentration (IC_50_) values of 4.26 and 4.63 µg/mL, respectively [6,8]. Since phenylalanine presented excellent bioactivity in other cyclic pentapeptides [9], we also incorporated phenylalanine into our extracted Galaxamide by replacing one of the leucines with phenylalanine. The obtained Galaxamide analog could inhibit the growth of HepG_2_, U87 and MCF-7 human carcinoma cell lines more efficiently than the natural Galaxamide [10]. Furthermore, we synthesized Galaxamide analogs by changing l-leucine to d*-*leucine; Galaxamide analogs with d*-*leucine exhibited stronger inhibition against human carcinoma cells than the natural Galaxamide. More recently, we demonstrated that more d*-*leucine incorporated in Galaxamide resulted in better antitumor activity; the analog with four d-leucines displayed the highest apoptosis effect against a wide range of human cancer cell lines, such as HepG2, MCF-7, SW480 and U87 [8].

**Figure 1:**
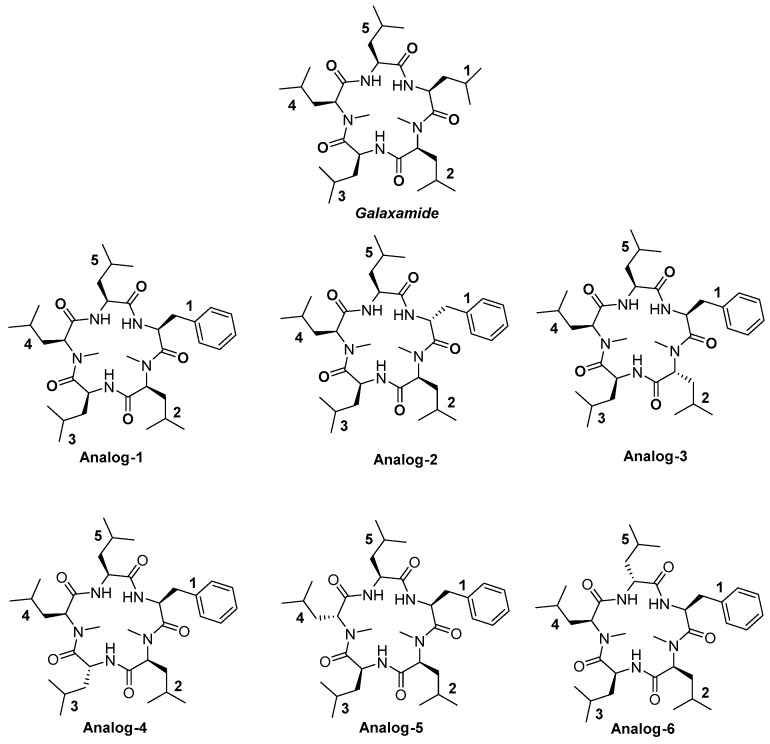
Chemical structure of Galaxamide and its analogs.

In the present work, we aimed to elucidate the relationship between the d-amino acid position in Galaxamide analogs (Figure 1) and their anticancer activity. For this purpose, we first synthesized the Galaxamide analog by replacing one leucine with phenylalanine and then gradually changed the d-amino acid position from phenylalanine to the other leucines to test the changes in their anticancer activity.

## 2. Results and Discussion

### 2.1. Synthesis of *Galaxamide* Analogs

**Scheme 1:**
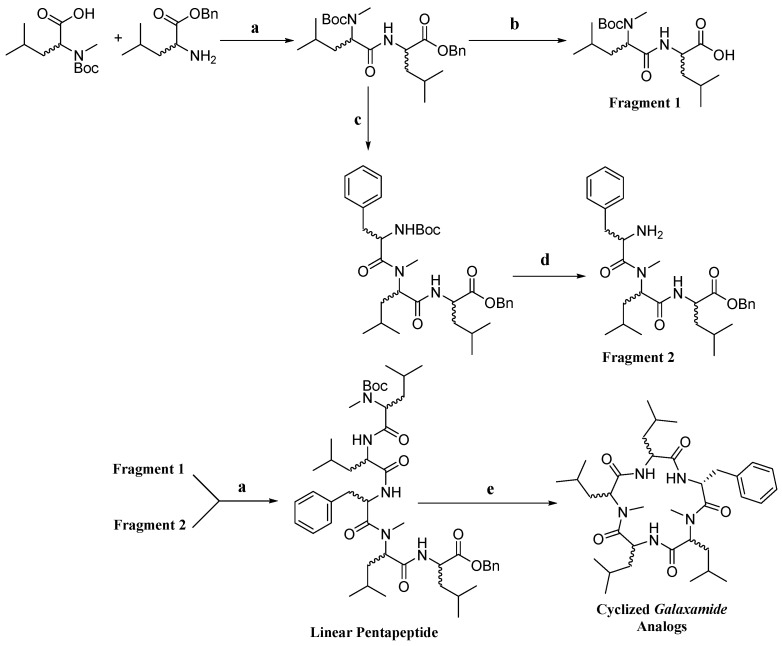
Synthesis of macrocycles. Reagents and conditions: a, DEPBT, DIEA, THF; b, H2, 10% Pd/C, EtOAc; c, (i) TFA, DCM; (ii) *N*-Boc- l(d)-Phe-OH, DEPBT, DIEA, THF; d, TFA, DCM; e, (i) TFA, DCM; (ii) H2, 10% Pd/C, EtOAc; (iii) DEPBT, HATU, TBTU. Note: DEPBT (3-(diethoxyphosphoryloxy)-1,2,3-benzotriazin-4(3H)-one), DIEA (*N*,*N*-Diisopropylethylamine), THF (tetrahydrofuran), EtOAc (ethyl acetate), TFA (trifluoroacetic acid), DCM (dichloromethane), TBTU (2-(1*H*-Benzotriazole-1-yl)-1,1,3,3-tetramethyluronium tetrafluoroborate), HATU (1-[Bis(dimethylamino)methylene]-1*H*-1,2,3-triazolo[4,5-b]pyridinium 3-oxid hexafluorophosphate).

**Figure 2:**
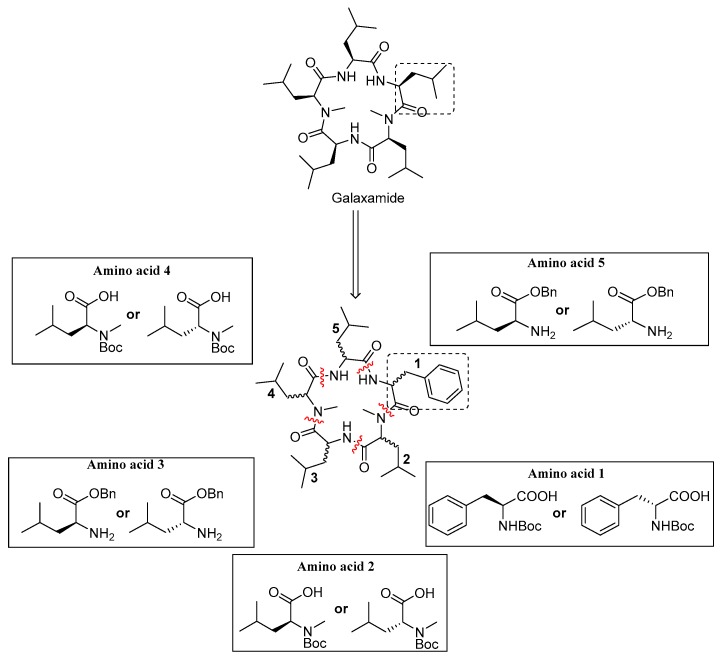
Amino acids used in the synthesis of Galaxamide analogs. Red wavy lines show the chemical bond where amino acid was replaced. Dash line boxes highlight leucine in Galaxamide has been replaced by phenylalanine in our Analogs. Amino acids in solid line boxes has d/l configuration.

In the present work, all of the Galaxamide analogs were designed and synthesized using the classic “3 + 2” strategy; the schematic illustration of the synthetic route is detailed in Scheme 1. As shown in Scheme 1, firstly, dipeptide was synthesized by the condensation reaction of *N*-Boc-Me-Leu-OH and l(d)-Leu-OBn·TosOH, where 3-(diethoxyphosphoryloxy)-1,2,3-benzotriazin-4(3*H*)-one (DEPBT) was used as the condensation reagent. After removing the C-terminal Bn group, the deprotected dipeptide was labeled as Fragment 1. Previous to the synthesis of tripeptide, the N-terminal Boc group was removed from the synthesized dipeptide. Then, the deprotected dipeptide was reacted with *N*-Boc-l(d)-phenylalanine to produce tripeptide. After removing the N-terminal Boc group, the deprotected tripeptide was marked as Fragment 2. The linear pentapeptide was synthesized through the condensation reaction of N-terminal protected dipeptide Fragment 1 and C-terminal protected tripeptide Fragment 2. To synthesize cyclic pentapeptide, the N-terminal Boc and C-terminal Bn groups were first removed from the obtained linear pentapeptide. Then, the deprotected linear pentapeptide was dissolved in a mixed solvent of THF/CH_3_CN/CH_2_Cl_2_ (2:2:1) with a final concentration of 0.1 M, and a mixture of condensation reagents of HATU/DEPBT/TBTU was added within the ice bath. The whole process was traced by High Performance Liquid Chromatography (HPLC), and the product was separated and purified by Reverse Phase-HPLC (RP-HPLC); finally, cyclic pentapeptides were obtained. General chemical structure of the synthesized Galaxamide analogs is shown in Figure 2.

### 2.2. Biological Activity

#### 2.2.1. Anticancer Activity of Galaxamide and Its Analogs on Different Human Cancer Cell Lines

To examine the anticancer potential of Galaxamide and its analogs, four different human cancer cell lines, such as human hepatocellular cells (HepG_2_), human breast cancer cells (MCF-7), human breast adenocarcinoma cells (MDA-MB-435) and a human cervical carcinoma cell line (Hela), were selected for the in vitro cancerous cell models. IC_50_ values of Galaxamide and its analogs on the four cell lines are shown in Table 1. Analog-1 and Analog-2 are Galaxamide analogs in which one of the leucines was replaced with phenylalanine, but varied in phenylalanine configuration (l-phenylalanine and d-phenylalanine for Analog-1, Analog-2, respectively). Generally, the incorporation of phenylalanine obviously improved the anticancer activity of Galaxamide, but with an exception for Hela cells. In particular, when l-phenylalanine was changed to d-phenylalanine, the inhibition activity of the Galaxamide analog was further reinforced to some extent, especially for MCF-7 cells. In MCF-7 cells, we found a 30% decrease in IC_50_ of Analog-2 (4.95 ± 0.18) compared to Analog-1 (13.03 ± 0.33), suggesting that d-amino acid can improve the anticancer effect of Galaxamide on cancer cells, which is consistent with our previous work [8].

On the basis of the obtained Galaxamide analog with one d-phenylalanine, we synthesized several other Galaxamide analogs by gradually changing the d-amino acid position from phenylalanine to other leucines. The anticancer activity of those analogs was also tested against the above-mentioned cell lines. Remarkably, varying the d-amino acid position greatly affected the anticancer activity of the analogs. Analog-6 with the d-amino acid at position 5 is the best among those analogs at inhibiting the growth of all of the cancer cells; the IC_50_ values were 4.1 ± 0.20 (HepG_2_), 8.15 ± 0.18 (MCF-7), 3.42 ± 0.25 (Hela) and 6.76 ± 0.22 (MDA-MB-435), which are nearly half those of the natural Galaxamide. Beside Analog-6, Analog-4 with the d-amino acid at position 3 also showed strong inhibition effect against HepG_2_, MCF-7, Hela and MDA-MB-435 with IC_50_ values of 7.2 ± 0.15, 8.62 ± 0.26, 4.2 ± 0.32, 7.67 ± 0.18, respectively. The anticancer ability of Analog-3 (d-amino acid at position 2) was only slightly changed compared to that of Galaxamide against HepG_2_, MCF-7, Hela and MDA-MB-435 with IC_50_ values of 7.71 ± 0.11, 18.16 ± 0.33, 4.39 ± 0.26, 12.6 ± 0.29, respectively, while Analog-5 (d-amino acid at position 2) displayed a decreased inhibition effect with IC_50_ values of 10.72 ± 0.32, 15.21 ± 0.29, 7.4 ± 0.33, 20.43 ± 0.23, respectively.

#### 2.2.2. Inhibition Effect of Galaxamide and Its Analogs on Cell Cycle

**Figure 3:**
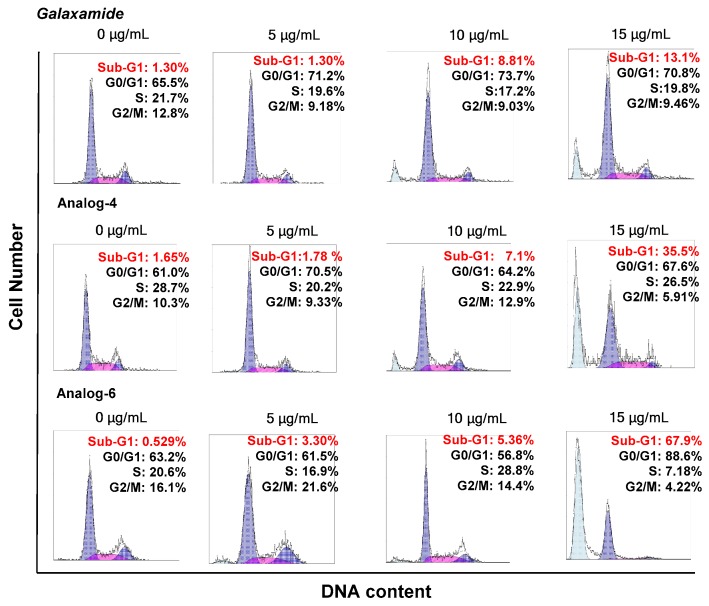
Cell cycle of HepG_2_ cells after 48 h of treatment with Galaxamide, Analog-4 and -6 with concentrations of 0, 5, 10 and 15 µg/mL. Sub-G1 (light blue peak), G0/G1 (light purple peak), S (pink peak) and G2/M (dark purple).

Since Analog-4 and -6 exhibited a better inhibition effect on the growth of cancer cells than the others, further investigation regarding their apoptotic mechanism was performed on HepG_2_. In particular, HepG_2_ cells were exposed to Galaxamide, Analog-4 and Analog-6 with concentrations of 0, 5, 10 and 15 µg/mL for 48 h. Then cell cycle of HepG_2_ cells was analyzed using the propidium iodide (PI) staining method, results are shown in Figure 3. Generally, treatment with Galaxamide, Analog-4 and -6 all caused changes in the cell cycle of HepG_2_ cells, mainly in the sub-G1 stage, where a subdiploid peak was detected, indicating their apoptotic effects on HepG_2_ cells. Moreover, a concentration-dependent apoptotic effect was observed, an increase in the concentration of Galaxamide, Analog-4 and -6 resulted in more apoptotic cells in the sub-G1 stage. Consistent with the above cytotoxicity evaluation, Analog-6 displayed the strongest apoptotic effects on HepG_2_ cells, where 68% of HepG_2_ cells were inhibited in the sub-G1 stage.

#### 2.2.3. Effect of Galaxamide and Analog-6 on the Cell Nuclear Integrity

We then investigated the effect of Galaxamide and Analog-6 on the cell morphology and nuclear integrity. HepG_2_ cells were treated with an increasing concentration of Analog-6 for 48 h and then stained by Hoechst 33342 (a DNA fluorescent dye). The effect of Galaxamide on HepG_2_ cells was used as a control. Noting that Hoechst 33342 is a DNA fluorescent dye, the fluorescently-stained DNA presents a blue color under the fluorescence microscope.

**Figure 4:**
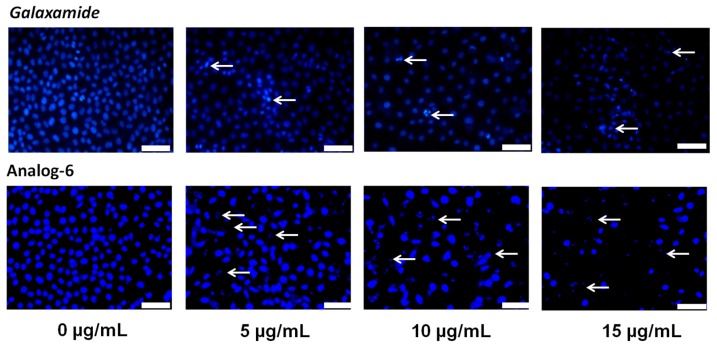
Fluorescent microscope images of HepG_2_ cells treated with different concentrations of Galaxamide and Analog-6 after Hoechst 33342 staining. White arrows in the each images indicate the cells with damaged nuclei (chromatin condensation and fragmentation). Scale bar is 50 μm.

In Figure 4, nuclei in the control cells show a regular round shape and are homogenously distributed inside the cells. Increasing the concentration of Galaxamide and Analog-6 caused a decrease in the number of nuclei and changes in the nuclei shape. For HepG_2_ cells exposed to Galaxamide, slight shrinking of nuclei was observed. However, the nuclei morphology of HepG_2_ cells was severely damaged at high concentrations of Analog-6, nuclei turned to being irregular and shrunk greatly, and the number of regular nuclei was largely decreased. Quantification of Hoechst data also shows that there is a significant difference in the percentage of cells with a regular nuclei shape between those treated with Analog-6 and Galaxamide, where the percentage in the Analog-6-treated group is much lower (Figure S1). The finding suggests that Analog-6 could cause obvious cell apoptosis, leading to the severe chromatin condensation.

#### 2.2.4. Apoptotic Mechanism Study of Galaxamide and Analog-6

**Figure 5:**
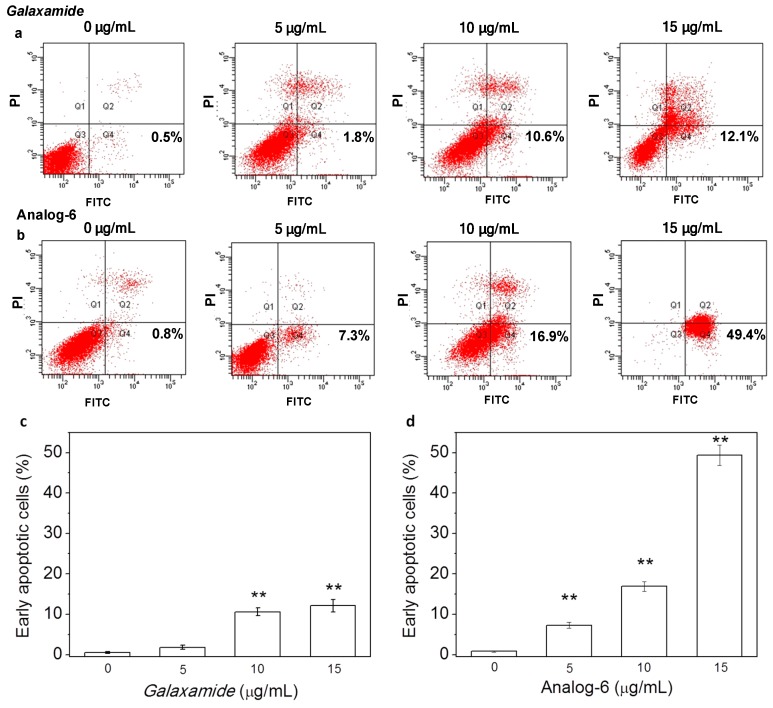
Flow cytometry results of HepG_2_ cells stained by Annexin V-fluorescein isothiocyanate (FITC) and propidium iodide (PI) after 48 h of exposure to 0, 5, 10 and 15 µg/mL Galaxamide (**a**) and Analog-6 (**b**); Comparison of the percentage of early apoptotic cells induced by Galaxamide (**c**) and Analog-6 (**d**); ** *p* < 0.01.

To further study the apoptotic mechanism of Analog-6, the Annexin V-fluorescein isothiocyanate (FITC) and propidium iodide (PI) dual staining method was used. Briefly, HepG_2_ cells were treated with 0, 5, 10 and 15 µg/mL Analog-6 for 48 h. Then, cells were washed several times with PBS and stained by dual staining of two fluorescent dyes, Annexin V-FITC and PI, and analyzed by flow cytometry. Results are shown in Figure 5, where HepG_2_ cells exposed to Galaxamide were used as the control. Note that Q1, Q2, Q3 and Q4 in the figure represent four quadrants of necrotic cells, late apoptotic cells, normal cells and early apoptotic cells, respectively.

Generally, exposure of Galaxamide and Analog-6 mainly caused the early apoptosis of HepG_2_ cells. Moreover, a dose-dependent apoptotic affect was observed in both cases, an increase in concentration led to a higher percentage of early apoptotic cells. In particular, when the Analog-6 concentration reached 15 µg/mL, almost 50% of the cells were found to be apoptotic in the early stage. In comparison, *Galaxamide* displayed a much weaker apoptotic effect with only 12.1% early apoptotic cells at a concentration of 15 µg/mL, further confirming the stronger cancer inhibition activity of Analog-6.

#### 2.2.5. Western Blot Analysis

**Figure 6:**
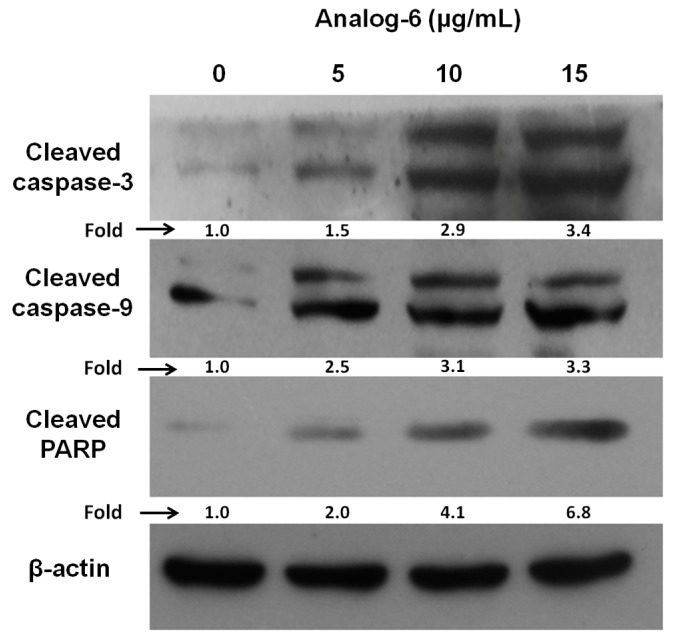
Western blot results display the expression of the cleaved caspase-3, caspase-9 and cleaved PARP of HepG_2_ cells after being treated with 0, 5, 10 and 15 µg/mL Analog-6 for 48 h.

There are two major pathways involved in cells apoptosis: the mitochondria-mediated intrinsic pathway and the death receptor-mediated extrinsic pathway [11]. To elucidate the possible mechanism of the apoptotic effects of Analog-6, Western blot experiments were performed. HepG_2_ cells were treated with 0, 5, 10 and 15 µg/mL Analog-6 for 48 h, and then expression of cleaved caspase-3, caspase-9 and poly(ADP-ribose) polymerase (PARP) was quantified, results are shown in Figure 6.

As was shown, an increase in the Analog-6 concentration resulted in the increasing expression of cleaved caspase-3, caspase-9 and PARP, suggesting that a mitochondria-mediated pathway was involved in the apoptotic process. At the concentration of 15 µg/mL, an increased fold of 3.4, 3.3 and 6.8 was found for cleaved caspase-3, caspase-9 and PARP, respectively. Therefore, Analog-6 exerted the early apoptotic effects on HepG_2_ cells through a mitochondria-mediated pathway, which is similar to the previous reports [9,12].

## 3. Discussion

In our previous work, we synthesized several Galaxamide analogs and tested their cytotoxicity on a normal cell line (human normal liver cell line L02) and a broad range of cancerous cell lines in vitro. It was revealed that Galaxamide and its analogs exhibited very low cytotoxicity on normal L02 (IC_50_ > 40 µg/mL) [8]. Moreover, the number of d-amino acids and the d-leucines greatly affected the anticancer behavior of Galaxamide analogs against in vitro cancerous cell lines. In particular, the analog containing four d-leucines presented the best anticancer potential. To explore a more potent anticancer agent, in the present work, we synthesized another six Galaxamide analogs (Analog-1 to Analog-6) and studied extensively the structure-activity relationship. Generally, when leucine in Galaxamide was replaced by phenylalanine (Analog-1 and Analog-2), the anticancer activity of Galaxamide was improved, suggesting that phenylalanine can act as an active site against cancer cells when incorporated in Galaxamide. Introduction of phenylalanine to sansalvamide A displayed similar results [9]. Besides, we found that the configuration of phenylalanine also matters for the anticancer activity of the Galaxamide analog. Analog-2 with d-phenylalanine showed a better inhibitory effect than Analog-1 with l-phenylalanine; our previous work also revealed a similar trend [8]. A possible mechanism is that the d-phenylalanine could more efficiently block the cyclic structure of Galaxamide, forming a more constrained confirmation, which could facilitate the stronger binding of Galaxamide analog to protein targets expressed by cancer cells [13]. Further change in the position of amino acid with the d configuration from phenylalanine to other leucines led to changes in the anticancer potential. Galaxamide analogs with d-amino acid in positions 3 and 5 (Analog-4 and Analog-6) displayed enhanced anticancer activity compared to the Galaxamide analog (Analog-2) with d-phenylalanine. In particular, Analog-6 demonstrated a two-fold elevated anticancer potential compared to Analog-2 on both HepG_2_ and MDA-MB-435 cell lines. This might be due to the more constrained and stable cyclic structure of Galaxamide achieved when d-amino acid was situated in positions 3 (Analog-4) and 5 (Analog-6), whereas Analog-6 presented the most stable cyclic configuration with the lowest energy and exhibited the most active amino acid residues for targeting cancer cells. Analog-3 with the d-amino acid in position 2 may have a similar structure-activity relationship as Analog-2, since their anticancer behavior is similar. The constrained structure of the Galaxamide analog may be destroyed to some extent when the d-amino acid was placed in position 4 (Analog-5), since Analog-5 displayed a decreased anticancer effect on the tested cancer cells.

Many cyclic-pentapeptides like RA-V (deoxybouvardin) and Sansalvamide A have presented great anticancer potential in cancer cells in vitro, mainly by triggering cell apoptosis. However, the exact apoptotic mechanism behind that is not clear yet. Sansalvamide A caused cell apoptosis through inhibiting the cell growth in the G0/G1 stage, while our synthesized Galaxamide analogs (Analog-4 and Analog-6) could inhibit HepG_2_ cell growth in the sub-G1 stage, suggesting a different apoptotic mechanism was involved. Further investigation of Analog-6 revealed that it caused the HepG_2_ cell apoptosis through a mitochondria-mediated pathway. By performing Western blot assays, we found a significant increase in the expression of cleaved caspase-3, caspase-9 and PARP, which is a sign of the activation of the cascade of the caspase family. This may be caused by the decrease of the mitochondrial membrane potential. A decrease in the potential is associated with changes in the mitochondrial membrane permeability, which can promote the entrance of the apoptotic proteins within the cytoplasm to the outer membrane and release of cytochrome C (Cyt-C) to cytoplasm [14]. The released Cyt-C in the cytoplasm then coupled with the apoptotic protease-activating factor 1 (Apaf-1), forming the Apaf-1/Cyt-C complex and initiating the activation of the cascade of the caspase family [15]. The activated caspase-9 activates the downstream caspase-3, which then activates endonuclease for degrading PARP, finally leading to cell apoptosis [16]. This suggests that Analog-6 exerted the early apoptotic effect through a mitochondria-mediated pathway.

## 4. Experimental Section

### 4.1. Synthesis of *Galaxamide* Analogs

Galaxamide analogues were synthesized following our reported protocols with some modification [6,8]. Briefly, a classic “3 + 2” synthetic strategy was used in the synthesis, and the whole process was performed in a liquid phase with *N-*Boc-l(d)-phenylalanine, *N*-Boc-l(d)-leucine and l(d)-Leu-OBn·TosOH as the starting materials. After sequential experimental steps of amine protection and deprotection, acid deprotection, methylation and condensation over amino acids, cyclization was finished using a combination of condensation reagents DEPBT, HATU and TBTU, leading to the product of cyclic pentapeptides.

#### 4.1.1. Synthesis of *N*-Boc-Leu-OH

In a 500-mL round-bottom flask under N_2_ atmosphere, 1 M NaOH solution (63 mL) in distilled water and 3.93 g l-Leu-OH (30 mmol) were added in sequence. The system was then immerged into the ice bath for cooling for 15 min. After the solution of l-Leu-OH become clear, 7.86 g Boc_2_O dissolved in THF (84 mL, 36 mmol) were added and stirred for 10 min; the pH was adjusted to around 10.5. The reaction temperature was then slowly increased to room temperature, and the solution was stirred overnight. After 24 h, the solvent was removed by vacuum distillation, and the rest was extracted twice using 200 mL ethyl ether. The resultant aqueous phase was cooled in the ice bath, acidified by adding an appropriate amount of 1 M HCl solution to increase the pH to 2. Then, the aqueous phase was further extracted three times with ethyl acetate (150 mL), washed twice with saturated NaCl solution (150 mL). Subsequently, the organic layer was dried by anhydrous sodium sulfate and filtered. After complete distillation of the organic solvent, 6.94 g white powder were obtained with a yield of 98.4%. ^1^H-NMR (500 MHz, CDCl_3_), *δ*_H_ 4.87 (m, 1H), 4.31 (m, 1H), 1.75 (m, 2H), 1.54 (m, 1H), 1.45 (s, 9H), 0.95 (d, 6H, *J* = 6.4 Hz); MS (ESI) *m*/*z*: 232.2 [M + H]^+^, 249.2 [M + NH_4_]^+^, 254.3 [M + Na]^+^.

#### 4.1.2. Synthesis of *N*-Boc-d-Leu-OH

The experimental procedure for the synthesis of *N*-Boc- d-Leu-OH followed as per described in the synthesis of *N*-Boc-Leu-OH. In this synthesis, 3.93 g d-Leu-OH were used instead of l-Leu-OH. As a result, 6.74 g white product were obtained with a yield of 97.2%. ^1^H-NMR (500 MHz, CDCl_3_), *δ*_H_ 4.87 (m, 1H), 4.31 (m, 1H), 1.75 (m, 2H), 1.54 (m, 1H), 1.45 (s, 9H), 0.95 (d, 6H, *J* = 6.4 Hz); MS (ESI) *m*/*z*: 232.2 [M + H]^+^, 249.2 [M + NH_4_]^+^, 254.3 [M + Na]^+^.

#### 4.1.3. Synthesis of *N*-Boc-Phe-OH

The experimental procedure for the synthesis of *N*-Boc-Phe-OH followed as per described in the synthesis of *N*-Boc-Leu-OH, and 4.95 g *L*-Phe-OH were used instead of 3.93 g l-Leu-OH. As a result, 7.72 g white product were obtained with a yield of 97%.

#### 4.1.4. Synthesis of *N*-Boc-d*-*Phe-OH

The experimental procedure for the synthesis of *N*-Boc-d*-*Phe-OH followed as per described in the synthesis of *N*-Boc-Leu-OH, and 4.95 g l-Phe-OH (30 mmol) were used instead of 3.93 g l-Leu-OH. As a result, 7.73 g white product were obtained with a yield of 97.2%.

#### 4.1.5. Synthesis of *N*-Boc-Me-Leu-OH

In a 250-mL round-bottom flask under N_2_ atmosphere, *N*-Boc-Leu-OH (4.62 g) and THF (60 mL) were added with stirring within the ice bath. After the solution of *N*-Boc-Leu-OH in THF become clear, CH_3_I (6.3 mL, 100 mmol) was added slowly and allowed to react for 30 min. Then, a 60% NaH dispersion in mineral oil (1.6 g, 40 mmol) was added slowly. The temperature of the system was increased slowly, and the solution was stirred overnight. After 24 h, an additional amount of 60% NaH in mineral oil (0.8 g, 20 mmol) was added after being cooled in the ice bath; again, the temperature increased slowly to room temperature, and it was stirred for overnight. After completion of the reaction, it was cooled in an ice bath, and distilled water (15 mL) was added slowly. Further, ethyl acetate (100 mL) was added to the flask under cooling in the ice bath. Then, the organic solvent was removed by vacuum distillation, and the residual was extracted twice using ethyl ether (60 mL). The combined ether phase was washed with saturated NaCl solution (50 mL) and combined with the aqueous phase. Under cooling in the ice bath, the solution was acidified by adding an appropriate amount of 1 M HCl to increase the pH to 2 to 3, and it was extracted three times using ethyl acetate (100 mL). Combining the organic phase together, it was washed by 150 mL 5% Na_2_S_2_O_3_ solution three times and saturated NaCl solution (100 mL). Subsequently, the solution was dried by anhydrous sodium sulfate, filtered, concentrated, dried under vacuum and purified using silica-gel column chromatography (eluant ratio of V*_petroleum ether_*/V*_acetone_* = 40:1); finally, 4.25 g white needle-like powder were obtained with a yield of 86.3%. m.p.: 62.6~64.3 °C, ${\left[\alpha \right]}_{D}^{26}+30.7{}^{\circ}$ (c = 0.5, EtOH), ^1^H-NMR (400 MHz, CDCl_3_), *δ*_H_ 4.82 (m, 1H), 4.62 (m, 1H), 3.34 (s, 3H), 1.71 (m, 2H), 1.58 (m, 1H), 1.46 (s, 9H), 0.95 (d, 6H, *J* = 6.4 Hz); MS (ESI) *m*/*z*: 246.2 [M + H]^+^, 263.2 [M + NH_4_]^+^, 268.3 [M + Na]^+^.

#### 4.1.6. Synthesis of *N*-Boc-Me-d-Leu-OH

The experimental procedure for the synthesis of *N*-Boc-Me-d-Leu-OH followed as per described in the synthesis of *N*-Boc-Me-Leu-OH, and *N*-Boc-d-Leu-OH (4.62 g) was used instead of *N*-Boc-Leu-OH. As a result, 4.62 g white powder product were obtained with a yield of 87.2%. ^1^H-NMR (500 MHz, CDCl_3_), *δ*_H_ 6.04 (m, 1H), 4.45 (m, 1H), 1.78 (m, 2H), 1.50 (m, 1H), 1.40 (s, 9H), 0.91 (d, 6H, *J* = 6.4 Hz); MS (ESI) *m*/*z*: 232.2 [M + H]^+^, 249.2 [M + NH_4_]^+^, 254.3 [M + Na]^+^.

#### 4.1.7. Synthesis of *N*-Boc-Me-Phe-OH

The synthesis followed the experimental ways for *N*-Boc-Me-Leu-OH as above. However, in this synthesis, 5.3 g *N*-Boc-*Phe*-OH were used instead of *N*-Boc-Leu-OH. As a result, 4.7 g colorless oily product were obtained with a yield of 85.0%. ^1^H-NMR (500 MHz, CDCl_3_), *δ*_H_ 7.35 (m, 5H), 7.02 (s, 1H), 4.90 (m, 1H), 3.57 (s, 3H), 3.12–3.43 (m, 1H), 1.42 (s, 9H); MS (ESI) *m*/*z*: 280.2 [M + H]^+^, 297.2 [M + NH_4_]^+^, 302.3 [M + Na]^+^.

#### 4.1.8. Synthesis of *N*-Boc-Me-d*-*Phe-OH

The experimental procedure for the synthesis of *N*-Boc-Me-Phe-OH followed as per described in the synthesis of *N*-Boc-Me-Leu-OH, and 5.3 g *N*-Boc-*D-Phe*-OH were used instead of 5.3 g *N*-Boc-Leu-OH.

#### 4.1.9. Synthesis of Dipeptide *N*-Boc-Me-Leu-Leu-OBn(D1)

In a 250-mL round-bottom flask under N_2_ atmosphere, 2.46 g *N*-Boc-Me-Leu-OH and 10 mL THF were added under stirring and cooled in ice bath condition. Then, DEPBT (4.5 g, 15 mmol) and DIEA (2.6 mL, 15 mmol) was added, and allowed to react for 10 min. After that 4.72 g l-Leu-OBn·TosOH (12 mmol) were added and stirred for complete dissolution. Then, the pH of reaction solution was adjusted to 7 to 8 by adding DIEA slowly. Subsequently, the temperature was increased slowly to room temperature, and the reaction was monitored using thin layer chromatography (TLC). After overnight stirring, the solvent was removed by vacuum distillation, and the residual crude product was purified directly using silica-gel column chromatography (eluant ratio of V*_petroleum ether_*/V*_acetone_* = 40:1). Finally, 3.76 g product were obtained with a yield of 83.8%. ^1^H-NMR (500 MHz, CDCl_3_), *δ*_H_ 7.35 (m, 5H), 7.02 (s, 1H), 4.90 (m, 1H), 3.57 (s, 3H), 3.12–3.43 (m, 1H), 1.42(s, 9H); MS (ESI) *m*/*z*: 280.2 [M + H]^+^, 297.2 [M + NH_4_]^+^, 302.3 [M + Na]^+^.

#### 4.1.10. Synthesis of Dipeptide *N*-Boc-Me-d-Leu-Leu-OBn(D2)

The experimental procedure for the synthesis of *N*-Boc-Me-d-Leu-Leu-OBn(D2) followed as per described in the synthesis of *N*-Boc-Me-Leu-Leu-OBn(D1). In this synthesis, *N*-Boc-Me-d-Leu-OH was used instead of *N*-Boc-Me-Leu-OH. As a result, 3.82 g product were obtained with a yield of 85.2%. ^1^H-NMR (300 MHz, CDCl_3_), *δ*_H_ 7.45–7.31 (m, 5H), 6.39 (d, *J* = 78.1 Hz, 1H), 5.24–5.11 (m, 2H), 4.65 (s, 2H), 2.76 (s, 3H), 1.69 (dd, *J* = 12.4, 6.4 Hz, 2H), 1.65–1.57 (m, 2H), 1.49 (s, 9H), 0.97–0.87 (m, 12H).; ^13^C-NMR (75 MHz, CDCl_3_), *δ*_C_ 172.5, 171.1, 167.4, 135.4, 128.6 (2C), 128.4, 128.2 (2C), 99.98, 67.0, 56.1, 50.7 (2C), 36.3, 29.8, 28.4 (3C), 24.8 (2C), 22.9 (2C), 22.1, 21.7; MS (ESI) *m*/*z*: 449.3 [M + H]^+^, 466.5 [M + NH_4_]^+^, 471.6 [M + Na]^+^.

#### 4.1.11. Synthesis of Dipeptide *N-*Boc-Me-Leu-d-Leu-OBn(D3)

The experimental procedure for the synthesis of *N-*Boc-Me-Leu-d-Leu-OBn(D3) followed as per described in the synthesis of *N*-Boc-Me-Leu-Leu-OBn(D1). In this synthesis, d-Leu-OBn·TosOH was used instead of l-Leu-OBn·TosOH. As a result, 3.92 g oily product were obtained with a yield of 87.4%. ^1^H-NMR (300 MHz, CDCl_3_), *δ*_H_ 7.42–7.29 (m, 5H), 6.64–6.31 (m, 1H), 5.14 (q, *J* = 12.3 Hz, 2H), 4.84–4.47 (m, 2H), 2.69 (s, 3H), 1.82–1.58 (m, 4H), 1.58–1.50 (m, 2H), 1.48 (s, 9H), 0.94 (t, *J* = 7.1 Hz, 12H); ^13^C-NMR (75 MHz, CDCl_3_), *δ*_C_ 172.6, 171.4, 157.1, 135.5, 128.6 (2C), 128.4, 128.2 (2C), 80.5, 66.9, 55.9, 50.8, 41.1, 36.0, 29.8, 28.3 (3C), 24.9, 24.7, 23.2, 22.8 (2C), 21.8; MS (ESI) *m*/*z*: 449.3 [M + H]^+^, 466.5 [M + NH_4_]^+^, 471.6 [M + Na]^+^.

#### 4.1.12. Synthesis of Dipeptide *N-*Boc-Me-Phe-Leu-OBn(D4)

The experimental procedure for the synthesis of *N-*Boc-Me-Phe-Leu-OBn(D4) followed as per described in the synthesis of *N*-Boc-Me-Leu-Leu-OBn(D1). In this synthesis, *N*-Boc-Me-Phe-OH was used instead of *N*-Boc-Me-Leu-OH. As a result, oily 3.98 g product were obtained with a yield of 82.6%. ^1^H-NMR (300 MHz, CDCl_3_), *δ*_H_ 7.45–7.30 (m, 5H), 7.25–7.17 (m, 5H), 5.37 (dd, *J* = 10.1, 5.3 Hz, 2H), 5.15 (q, *J* = 12.3 Hz, 2H), 4.85 (dd, *J* = 15.6, 6.7 Hz, 1H), 3.13–2.98 (m, 1H), 2.86 (s, 3H), 2.84–2.77 (m, 1H), 1.87–1.60 (m, 2H), 1.47–1.39 (m, 9H), 1.38–1.24 (m, 2H), 0.92 (dd, *J* = 8.4, 6.7 Hz, 6H); ^13^C-NMR (75 MHz, CDCl_3_), δ_C_ 172.7, 171.3, 155.3, 136.3, 135.6, 129.5 (2C), 129.4, 128.6 (2C), 128.4 (2C), 128.3 (2C), 126.8, 79.6, 77.2, 66.9, 54.8, 51.7, 38.7, 37.1, 28.0 (3C), 24.7, 23.3, 21.5; MS (ESI) *m*/*z*: 483.7 [M + H]^+^, 500.6 [M + NH_4_]^+^, 505.6 [M + Na]^+^.

#### 4.1.13. Synthesis of Dipeptide *N-*Boc-d-Phe-Me-Leu-OBn(D5)

The experimental procedure for the synthesis of *N-*Boc-d-Phe-Me-Leu-OBn(D5) followed as per described in the synthesis of *N*-Boc-Me-Leu-Leu-OBn(D1). In this synthesis, *N*-Boc-Me-d-Phe-OH was used instead of *N*-Boc-Me-Phe-OH. As a result, oily 4.05 g product were obtained with a yield of 84.1%. ^1^H-NMR (300 MHz, CDCl_3_), *δ*_H_ 7.46–7.33 (m, 5H), 7.32–7.16 (m, 5H), 6.48 (dd, *J* = 95.7, 7.5 Hz, 1H), 5.17 (q, *J* = 12.3 Hz, 2H), 4.84 (ddd, *J* = 54.7, 17.6, 8.4 Hz, 2H), 3.54–3.27 (m, 1H), 3.01–2.84 (m, 1H), 2.76 (s, 3H), 1.74–1.60 (m, 1H), 1.54 (dd, *J* = 14.7, 7.7 Hz, 2H), 1.34 (s, 9H), 0.90 (d, *J* = 5.4 Hz, 6H); ^13^C-NMR (75 MHz, CDCl_3_), *δ*_C_ 172.5, 170.6, 156.7, 137.5, 135.4, 129.1, 129.0, 128.6 (2C), 128.4 (2C), 128.2, 126.4, 80.5, 67.0, 59.4, 50.8, 41.2, 33.9, 28.2 (3C), 25.0, 24.8, 22.8, 21.8; MS (ESI) *m*/*z*: 483.7 [M + H]^+^, 500.6 [M + NH_4_]^+^, 505.6 [M + Na]^+^.

#### 4.1.14. Synthesis of Tripeptide *N-*Boc-Leu-Me-Leu-Leu-OBn(T1)

D_1_ (3.14g, 7 mmol) was first dissolved in 32 mL dichloromethane, and then, 1.47 mL anisole were added under N_2_ protection. The solution was cooled in the ice bath and stirred continuously; TFA (8 mL) was added slowly to the solution and allowed to react for 4 h. The solvent was removed by vacuum distillation, then additional appropriate dichloromethane was added and vacuum distillated again. This step was repeated three times for complete removal of TFA; finally, an oily product named as D_2_-A was obtained after drying, and it was used for further synthesis.

BOC-Leu-OH (1.85 g, 8 mmol) was dissolved in THF (5 mL) under stirring and cooled in an ice bath in N_2_ atmosphere. Subsequently, DEPBT (3.00 g, 10 mmol) and DIEA (1.8 mL, 10 mmol) were added into the solution and reacted for 10 min. Thereafter, an appropriate amount of the previously synthesized D_2_-A THF solution (10 mL) was added, and DIEA was used to adjust the pH of the solution to 7 to 8. The temperature was slowly increased to room temperature, and the reaction was monitored by TLC. After completion of the reaction, the solvent was removed by vacuum distillation, and the residual crude product was purified directly using silica-gel column chromatography (eluant ratio of V*_petroleum ether_*/V*_acetone_* = 40:1). Finally, 2.95 g colorless oily product were obtained with an overall yield of 80.8%. ^1^H-NMR (500 MHz, CDCl_3_), *δ*_H_ 7.32 (m, 5H), 6.32 (d, 1H, *J* = 4.0 Hz), 5.09 (m, 4H), 4.56 (m, 2H), 2.94 (s, 3H), 1.63 (m, 3H), 1.46 (m, 3H), 1.41 (s, 9H), 1.23 (m, 3H), 0.90 (m, 18H); ^13^C-NMR (125 MHz, CDCl_3_), *δ*_C_ 174.1, 172.3, 170.2, 155.7, 135.3, 128.5, 128.4, 128.3, 128.2, 127.9, 79.6, 67.0, 54.3, 50.6, 49.0, 41.9, 40.9, 35.9, 30.1, 28.3 (3C), 24.7, 24.6, 23.4 (2C), 22.9, 22.8, 22.0 21.6,21.5; MS (ESI) *m*/*z*: 562.8 [M + H]^+^, 579.8 [M + NH_4_]^+^, 584.9 [M + Na]^+^.

#### 4.1.15. Synthesis of Tripeptide *N-*Boc-Leu-Me-Leu-d*-*Leu-OBn(T2)

The experimental procedure for the synthesis of *N-*Boc-Leu-Me-Leu-d*-*Leu-OBn(T2) followed as per described in the synthesis of *N*-Boc-Leu-Me-Leu-Leu-Obn(T1). In this synthesis, D3 was used instead of D1. ^1^H-NMR (300 MHz, CDCl_3_), *δ*_H_ 7.40–7.32 (m, 5H), 5.20–5.19 (m, 1H), 5.10 (dd, *J* = 12.6, 5.5 Hz, 1H), 4.60–4.56 (m, 1H), 2.90 (s, 3H), 1.70–1.63 (m, 6H), 1.42 (s, 9H), 1.35 (d, *J* = 10.8 Hz, 3H), 0.97–0.90 (m, 18H); ^13^C-NMR (75 MHz, CDCl_3_), *δ*_C_ 174.9, 172.4, 170.6, 155.7, 135.5, 128.5 (2C), 128.5, 128.3 (2C), 128.2, 79.6, 66.9, 54.2, 50.7, 49.2, 41.6, 40.9, 35.6, 30.2, 28.3 (3C), 24.8, 24.6, 23.4, 23.1, 22.8, 22.0, 21.7, 21.6; MS (ESI) *m*/*z*: 562.8 [M + H]+, 579.8 [M + NH_4_]^+^, 584.9 [M + Na]^+^.

#### 4.1.16. Synthesis of Tripeptide *N-*Boc-Phe-Me-Leu-d*-*Leu-OBn(T3)

The experimental procedure for the synthesis of *N-*Boc-Phe-Me-Leu-d*-*Leu-OBn(T3) followed as per described in the synthesis of *N*-Boc-Leu-Me-Leu-Leu-Obn(T1). In this synthesis, Boc-Phe-OH was used instead of Boc-Leu-OH. ^1^H-NMR (300 MHz, CDCl_3_), *δ*_H_ 7.37–7.33 (m, 5H), 7.32–7.30 (m, 1H), 7.28–7.19 (m, 5H), 6.20 (d, *J* = 8.3 Hz, 1H), 5.15–5.03 (m, 2H), 4.81 (dt, *J* = 9.1, 6.6 Hz, 1H), 4.65–4.52 (m, 2H), 3.09 (dd, *J* = 13.8, 6.6 Hz, 1H), 2.96–2.85 (m, 01H), 2.81 (s, 3H), 1.95–1.71 (m, 2H), 1.58–1.46 (m, 2H), 1.39 (s, 9H), 1.30 (m, 2H), 0.95 (t, *J* = 5.4 Hz, 9H), 0.80 (dd, *J* = 6.6, 1.4 Hz, 3H); MS (ESI) *m*/*z*: 595.7 [M + H]^+^, 612.7 [M + NH_4_]^+^, 617.9 [M + Na]^+^.

#### 4.1.17. Synthesis of Tripeptide *N-*Boc-Phe-Me-Leu*-*Leu-OBn(T4)

The experimental procedure for the synthesis of *N-*Boc-Phe-Me-Leu*-*Leu-OBn(T4) followed as per described in the synthesis of *N*-Boc-Leu-Me-Leu-Leu-OBn(T1). In this synthesis, Boc-Phe-OH was used instead of Boc-Leu-OH. ^1^H-NMR (300 MHz, CDCl_3_), *δ*_H_ 7.93 (d, *J* = 7.9 Hz, 1H), 7.42–7.32 (m, 5H), 7.32–7.18 (m, 5H), 6.18 (d, *J* = 8.0 Hz, 1H), 5.31–5.20 (m, 1H), 5.20–5.13 (m, 2H), 4.85–4.71 (m, 1H), 4.71–4.55 (m, 1H), 3.18–2.99 (m, 1H), 2.90–2.81 (m, 1H), 2.79 (s, 3H), 1.88 (dd, *J* = 9.5, 3.6 Hz, 1H), 1.76–1.66 (m, 1H), 1.66–1.57 (m, 2H), 1.57–1.44 (m, 2H), 1.40 (d, *J* = 1.6 Hz, 9H), 0.98–0.76 (m, 12H); ^13^C-NMR (75 MHz, CDCl_3_), *δ*_C_ 173.2, 172.3, 170.2, 155.2, 135.4, 129.3, 128.9, 128.6 (2C), 128.5, 128.4, 128.3 (2C), 128.2, 128.0, 127.0, 79.9, 67.1, 55.0, 50.8, 41.1, 39.0, 36.4, 30.6, 28.3 (3C), 24.9, 24.7 (2C) 23.1, 22.8, 22.0, 21.9; MS (ESI) *m*/*z*: 595.7 [M + H]^+^, 612.7 [M + NH_4_]^+^, 617.9 [M + Na]^+^.

#### 4.1.18. Synthesis of Tripeptide *N-*Boc-Phe-Me-d-Leu-Leu-OBn(T5)

The experimental procedure for the synthesis of *N-*Boc-Phe-Me-d-Leu-Leu-OBn(T5) followed as per described in the synthesis of *N*-Boc-Leu-Me-Leu-Leu-OBn(T1). In this synthesis, D2 was used instead of D1. ^1^H-NMR (300 MHz, CDCl_3_), *δ*_H_ 7.41–7.33 (m, 5H), 7.33–7.20 (m, 5H), 6.89 (d, *J* = 7.4 Hz, 1H), 5.25 (d, *J* = 6.8 Hz, 1H), 5.21–5.13 (m, 2H), 5.08 (d, *J* = 12.2 Hz, 1H), 4.71 (dd, *J* = 14.8, 6.8 Hz, 1H), 4.66–4.57 (m, 1H), 3.04–2.94 (m, 1H), 2.60 (s, 3H), 1.79–1.67 (m, 2H), 1.55 (dd, *J* = 12.9, 5.8 Hz, 2H), 1.43 (s, 9H), 1.39–1.24 (m, 2H), 0.91 (dd, *J* = 6.0, 2.9 Hz, 6H), 0.86 (d, *J* = 6.5 Hz, 6H); MS (ESI) *m*/*z*: 95.7 [M + H]^+^, 612.7 [M + NH_4_]^+^, 617.9 [M + Na]^+^.

#### 4.1.19. Synthesis of Linear Pentapeptide *N*-Boc-Me-Leu-Leu-Phe-Me-d-Leu-Leu-OBn(P1)

In a 50-mL round-bottom flask with N_2_ protection, D1 (1.68 g) was dissolved in ethyl acetate (20 mL), and then 10% catalyst Pd/C catalyst (0.56 g) was added. The flask was sealed and flushed with H_2_, the solution was stirred at room temperature and monitored for the progress of the reaction by TLC. After 4–6 h of reaction when the TLC point of the raw materials disappeared, H_2_ flow was closed, and the reaction was stopped. The solution was first filtered to recycle the catalyst Pd/C, and the rest of the solution was distillated in a vacuum to remove the solvent residues and was dried in the oven. Finally, the colorless product of D1-B was obtained and used for the following step.

The previously-obtained product T5 (1.61 g) was dissolved in CH_2_Cl_2_ (20 mL) under N_2_ atmosphere, then anisole (0.76 mL, 7.2 mmol) was added under stirring and cooled in an ice bath. TFA (8 mL) was added to the solution slowly and stirred for reaction 4 h. Thereafter, the solvent was removed by vacuum distillation, then additional appropriate dichloromethane was added and distillated under vacuum again to remove traces of TFA. This step was repeated three times for complete removal of TFA. Finally, the oily product of T5-A was obtained for the next step.

The above obtained T5-A was dissolved in 5 mL THF under stirring and cooled in an ice bath. Subsequently, DEPBT (2.15 g, 7.2 mmol) and DIEA (1.26 mL, 7.2 mmol) were added into the solution and reacted for 10 min Thereafter, an appropriate amount of the above obtained D1-B THF solution (5 mL) was added, and DIEA was used to adjust the pH of the solution to 7–8. The temperature was increased slowly to room temperature and monitored for the progress of reaction by TLC. The solution was stirred 24 h; the solvent was removed by vacuum distillation; and the residual crude product was purified directly using silica-gel column chromatography (eluant ratio of V*_petroleum ether_*/V*_acetone_* = 20:1). ^1^H-NMR (300 MHz, CDCl_3_), *δ*_H_ 7.31 (m, 7H), 7.23 (m, 3H), 6.76 (d, *J* = 8.4 Hz, 1H), 6.36 (d, *J* = 8.4 Hz, 1H), 5.30 (d, *J* = 8.4 Hz, 1H), 5.13 (s, 2H), 5.01 (m, 1H), 4.83 (m, 2H), 4.70 (m, 1H), 4.56 (m, 1H), 2.96 (s, 3H), 2.73 (m, 2H), 2.67 (s, 3H), 1.64 (m, 2H), 1.59 (m, 6H), 1.49 (m, 4H), 1.41 (s, 9H), 0.91 (m, 24H); ^13^C-NMR (75 MHz, CDCl_3_), *δ*_C_ 172.62, 172.5, 171.8, 169.8, 169.5, 137.7, 128.9, 128.8, 128.2 (2C), 128.1, 128.1, 128.0, 128.0, 127.8 (2C), 127.5, 126.6, 76.8, 66.6, 66.2, 54.2, 54.1, 50.2, 47.2, 40.8, 40.7, 39.5, 39.3, 35.5, 30.0, 29.9, 27.9 (3C), 24.4, 24.3, 24.3, 24.2, 22.8, 22.6, 22.5, 22.4, 21.7, 21.6, 21.2, 21.1; MS (ESI) *m*/*z*: 836.9 [M + H]^+^, 853.9 [M + NH_4_]^+^, 858.9 [M + Na]^+^.

#### 4.1.20. Synthesis of Linear Pentapeptide *N*-Boc-Me-Leu-Leu-Phe-Me-Leu-d-Leu-OBn(P2)

The experimental procedure was followed as per described for the synthesis of P1. In this synthesis, T3 and T3-A were used instead of T5 and T5-A in the corresponding places, respectively. ^1^H-NMR (300 MHz, CDCl_3_), *δ*_H_ 7.36–7.27 (m, 5H), 7.27–7.12 (m, 5H), 6.94 (d, *J* = 8.3 Hz, 1H), 6.55 (d, *J* = 27.8 Hz, 2H), 5.14 (s, 1H), 5.15–5.09 (m, 2H), 5.04 (dd, *J* = 19.1, 6.7 Hz, 1H), 4.72–4.54 (m, 2H), 4.45 (dd, *J* = 18.2, 10.1 Hz, 1H), 3.30–3.05 (m, 1H), 2.98–2.87 (m, 1H), 2.85 (s, 3H), 2.71 (s, 3H), 1.92–1.52 (m, 8H), 1.48 (d, *J* = 4.5 Hz, 9H), 1.39–1.23 (m, 4H), 0.99–0.74 (m, 24H); MS (ESI) *m*/*z*: 836.7 [M + H]^+^, 853.8 [M + NH_4_]^+^, 858.7 [M + Na]^+^.

#### 4.1.21. Synthesis of Linear Pentapeptide *N*-Boc-Me-d-Leu-Leu-Phe-Me-Leu-Leu-OBn(P3)

The experimental procedure was followed as per described for the synthesis of P1. In this synthesis, D2 and D2-B were used instead of D1 and D1-B, and T4 and T4-A were used instead of T5 and T5-A in the corresponding places, respectively. ^1^H-NMR (300 MHz, CDCl_3_), *δ*_H_ 7.46–7.33 (m, 5H), 7.20 (dt, *J* = 12.2, 7.3 Hz, 5H), 7.05 (d, *J* = 8.1 Hz, 1H), 6.23 (d, *J* = 4.7 Hz, 1H), 5.70 (dd, *J* = 11.8, 5.2 Hz, 1H), 5.27–5.16 (m, 2H), 5.16–5.09 (m, 1H), 4.77–4.56 (m, 2H), 4.31 (dd, *J* = 13.0, 5.3 Hz, 1H), 3.63–3.46 (m, 1H), 2.92 (s, 3H), 2.84 (s, 3H), 2.82–2.71 (m, 1H), 2.10–1.61 (m, 8H), 1.44 (s, 9H), 1.26 (dd, *J* = 15.0, 5.5 Hz, 1H), 1.02 (d, *J* = 6.4 Hz, 3H), 1.00–0.92 (m, 12H), 0.91 (d, *J* = 1.9 Hz, 6H), 0.74–0.58 (m, 6H); ^13^C-NMR (75 MHz, CDCl_3_), *δ*_C_ 174.9, 173.3 172.7, 171.6, 170.3, 155.7, 137.3, 135.6, 128.8, 128.6 (2C), 128.5 (2C), 128.4 (2C), 128.3 (2C), 126.5, 79.7, 66.9, 56.9, 54.8, 51.0, 49.2, 48.3, 41.6, 40.4, 40.3, 35.3, 33.6, 30.9, 30.4, 28.3 (3C), 24.8 (2C), 24.6, 24.5, 24.1, 23.4, 23.3, 22.9 (2C), 22.1, 21.7, 21.4; MS (ESI) *m*/*z*: 836.7 [M + H]^+^, 853.8 [M + NH_4_]^+^, 858.7 [M + Na]^+^.

#### 4.1.22. Synthesis of Linear Pentapeptide *N*-Boc-Me-Leu-d*-*Leu-Phe-Me-Leu-Leu-OBn(P4)

The experimental procedure was followed as per described for the synthesis of P1. In this synthesis, D3 and D3-A were used instead of D1and D1-A, and T4 and T4-B were used instead of T5 and T5-B in the corresponding places, respectively. ^1^H-NMR (300 MHz, CDCl_3_), *δ*_H_ 7.36 (dt, *J* = 11.0, 3.6 Hz, 5H), 7.30–7.20 (m, 5H), 6.48 (d, *J* = 8.6 Hz, 1H), 6.38 (d, *J* = 8.1 Hz, 1H), 5.30 (d, *J* = 9.1 Hz, 1H), 5.24–5.15 (m, 2H), 5.14–5.03 (m, 2H), 5.00–4.89 (m, 1H), 4.84 (d, *J* = 9.0 Hz, 1H), 4.63–4.49 (m, 1H), 3.11–2.95 (m, 1H), 2.93 (s, 3H), 2.86 (s, 3H), 2.82–2.67 (m, 1H), 1.81–1.50 (m, 8H), 1.46 (d, *J* = 7.8 Hz, 4H), 1.40 (s, 9H), 1.08–0.73 (m, 24H); ^13^C-NMR (75 MHz, CDCl_3_), *δ*_C_ 173.5, 173.1, 172.4, 170.5, 170.2, 155.2, 135.5, 134.0, 129.3, 128.7 (2C), 128.6 (3C), 128.4, 128.2 (2C), 127.0, 79.8, 66.9, 55.0, 54.3, 51.9, 50.7, 47.7, 41.4, 41.0, 39.0, 35.5, 30.6, 30.2, 28.4, 28.3 (3C), 24.9, 24.8, 24.6, 23.4, 23.1, 23.1, 22.9, 22.8, 22.0, 22.0, 21.7, 21.6; MS (ESI) *m*/*z*: 836.7 [M + H]^+^, 853.8 [M + NH_4_]^+^, 858.7 [M + Na]^+^.

#### 4.1.23. Synthesis of Linear Pentapeptide *N*-Boc-Leu-Me-Leu-Leu-Me-Phe-Leu-OBn(P5)

The experimental procedure was followed as per described for the synthesis of P1. In this synthesis, T4 and T4-A were used instead of T5 and T5-A in the corresponding places, respectively. ^1^H-NMR (300 MHz, CDCl_3_), *δ*_H_ 8.55–8.15 (m, 1H), 7.43–7.33 (m, 5H), 7.32–7.17 (m, 5H), 7.15–7.09 (m, 1H), 5.19 (dd, *J* = 15.3, 2.9 Hz, 1H), 5.15–5.11 (m, 1H), 4.90–4.70 (m, 1H), 4.69–4.53 (m, 2H), 4.42–4.19 (m, 1H), 3.35–3.20 (m, 1H), 3.07 (dd, *J* = 18.9, 8.5 Hz, 1H), 2.95 (s, 3H), 2.77 (s, 3H), 1.81–1.65 (m, 8H), 1.44 (s, 9H), 1.32–1.22 (m, 2H), 0.99–0.48 (m, 24H); ^13^C-NMR (75 MHz, CDCl_3_), *δ*_C_ 174.1, 172.9, 172.8, 171.4, 169.4, 155.8, 137.9, 135.8, 129.6, 129.2 (2C), 128.9 (2C), 128.6 (2C), 128.2, 128.0, 127.0, 77.2, 67.0, 66.8, 62.7, 53.5, 51.5, 49.0, 47.0, 42.0, 39.9, 35.4, 34.0, 29.9, 29.5, 28.3 (3C), 24.8, 24.6 (2C), 24.3, 23.9, 23.5, 23.4, 23.1, 22.9, 21.7, 21.2, 19.9; MS (ESI) *m*/*z*: 836.7 [M + H]^+^, 853.8 [M + NH_4_]^+^, 858.7 [M + Na]^+^.

#### 4.1.24. Synthesis of Linear Pentapeptide *N*-Boc-Leu-Me-Leu-Leu-Me-d*-*Phe-Leu-OBn(P6)

The experimental procedure was followed as per described for the synthesis of P1. In this synthesis, D1 and D1-A were used instead of T1 and T1-A, and T5 and T5-B were used instead of D5 and D5-B in the corresponding places, respectively. ^1^H-NMR (500 MHz, CDCl_3_), *δ*_H_ 7.31 (m, 7H), 7.26–7.23 (m, 3H), 6.76 (d, 1H, *J* = 10.0 Hz), 6.36 (d, 1H, *J* = 10.0 Hz), 5.30 (d, 1H, *J* = 10.0 Hz), 5.13 (s, 2H), 5.05–5.01 (m, 1H), 4.90–4.83 (m, 2H), 4.73–4.70 (m, 1H), 4.59–4.56 (m, 1H), 2.96 (s, 3H), 2.77–2.73 (m, 2H), 2.67 (s, 3H), 1.67–1.64(m, 2H), 1.62–1.67 (m, 6H), 1.50–1.46 (m, 4H), 1.41 (s, 9H), 0.98–088 (m, 24H), MS (ESI) *m*/*z*: 837.0 [M + H]^+^, 854.0 [M + NH_4_]^+^, 859.0 [M + Na]^+^.

#### 4.1.25. Synthesis of Cyclo(Me-Leu-Leu-Phe-Me-Leu-Leu) (Analog-1)

P5 (0.12 g, 0.15 mmol) was first dissolved in dichloromethane (20 mL) in a 50-mL round-bottom flask, and then, TFA (5 mL) was added under stirring and cooled in an ice bath. After the reaction was stirred for 4 h, the solvent was removed by vacuum distillation, then additional appropriate dichloromethane was added and distillated under vacuum again. This step was repeated three times for complete removal of TFA; finally, an oily product was obtained after drying. Then, the obtained oily product was dissolved in ethyl acetate (20 mL) under N_2_ atmosphere, and 10% Pd/C (0.2 g) catalyst was added. The flask was sealed and flushed with H_2_; the solution was stirred under room temperature and monitored for the progress of reaction by TLC. After 4–6 h of reaction when the TLC point of the raw materials disappeared, H_2_ flow was closed, and the reaction was stopped. The solution was first filtered to recycle the catalyst Pd/C, and the rest of the solution was distillated under vacuum to remove the solvent residues and was dried in the oven. Finally, a colorless product was obtained. The as-obtained product was then dissolved in 50 mL THF/CH2Cl2/CH3CN (2:1:2) mixed solvent under N_2_ atmosphere; 0.067 g HATU, 0.065 g DEPBT and 0.07 g TBTU were added in sequence and allowed to react for 4 days. The solvent was removed by vacuum distillation, and the solid product was dissolved in CH_2_Cl_2_ and washed by saturated NaHCO_3_ and H_2_O separately. The resultant organic layer was dried by anhydrous Na_2_SO_4_ and concentrated, then purified by RP-HPLC (Agilent Eclipse ZORBAX SB-C18, 5 µm, 9.4 × 250 nm). Finally, 11.2 mg white powder with a yield of 40.2% were obtained. ^1^H-NMR (300 MHz, CDCl_3_), *δ*_H_ 8.15 (d, *J* = 9.7 Hz, 1H), 8.15 (d, *J* = 9.7 Hz, 1H), 7.53 (d, *J* = 8.7 Hz, 1H), 7.53 (d, *J* = 8.7 Hz, 1H), 7.36–7.25 (m, 3H), 7.18–7.11 (m, 2H), 5.80 (d, *J* = 7.3 Hz, 1H), 4.86–4.72 (m, 2H), 4.27 (ddd, *J* = 11.1, 7.3, 3.6 Hz, 1H), 3.67 (dd, *J* = 10.3, 5.8 Hz, 1H), 3.67 (dd, *J* = 10.3, 5.8 Hz, 1H), 3.45 (d, *J* = 7.8 Hz, 1H), 3.39 (dd, *J* = 8.7, 6.4 Hz, 1H), 3.12 (dd, *J* = 12.9, 5.8 Hz, 1H), 2.95 (s, 3H), 2.81 (s, 3H), 2.32 (ddd, *J* = 14.2, 8.7, 5.7 Hz, 1H), 2.02–1.90 (m, 1H), 1.90–1.73 (m, 4H), 1.60–1.55 (m, 1H), 1.50–1.32 (m, 4H), 1.26–1.18 (m, 1H), 0.95–0.91 (m, 9H), 0.89 (d, *J* = 4.7 Hz, 9H), 0.86 (s, 3H), 0.83 (d, *J* = 6.5 Hz, 3H); ^13^C-NMR (75 MHz, CDCl_3_), *δ*_C_ 174.5, 173.0, 172.6, 171.2, 169.7, 136.5 (2C), 129.2 (2C), 129.1, 127.6, 71.1, 68.0, 53.3, 49.3, 48.1, 41.5, 41.1, 40.7, 40.0, 39.6, 38.8, 34.0, 26.1, 25.2, 25.1, 25.0, 23.6, 23.4, 23.2, 23.1, 22.6, 22.1, 21.8, 21.3; MS (ESI) *m*/*z*: 628.8 [M + H]^+^, 645.8 [M + NH_4_]^+^, 650.8 [M + Na]^+^.

For the detailed characterization of mass spectra, mass-mass spectra, ^1^H-NMR and HPLC regarding Analog-1, refer to Figures S2A, S3A, S4A and S5A.

#### 4.1.26. Synthesis of Cyclo(Me-Leu-Leu-d-Phe-Me-Leu-Leu) (Analog-2)

The experimental procedure was followed as described in the synthesis of Analog-1. In this synthesis, P6 was used instead of P5. Finally, 31.8 mg white powder with a yield of 42.3% were obtained. ^1^H-NMR (500 MHz, CDCl_3_), *δ*_H_ 8.13 (s, 1H), 7.74–7.71 (m, 1H), 7.29–7.19 (m, 5H), 6.91 (d, *J* = 10.0 Hz, 1H), 5.18 (t, *J* = 10.0Hz, 1H), 4.91–4.87 (m, 1H), 4.79–4.74 (m, 3H), 3.09–3.05 (m, 2H), 2.94–2.91 (m, 1H), 2.84 (s, 1H), 2.80 (s, 2H), 2.62 (s, 3H), 1.93–1.88 (m, 1H), 1.82–1.77 (m, 1H), 1.61–1.50 (m, 6H), 1.43–1.37 (m, 1H), 1.12–1.06 (m, 1H), 0.97–0.89 (m, 18H), 0.73–0.66 (m, 6H); ^13^C-NMR (125 MHz, CDCl_3_), *δ*_C_ 175.9, 171.7, 171.5, 170.1, 170.0, 136.2, 129.1 (2C), 128.8 (2C), 127.2, 57.8, 53.7 (2C), 48.7, 47.8, 41.5, 41.1, 37.7, 37.5, 34.5, 31.4, 29.5, 24.9 (2C), 24.8, 24.5, 23.2, 23.1, 22.9 (2C), 22.2, 21.9, 21.8, 21.6; MS (ESI) *m*/*z*: 628.8 [M + H]^+^, 645.9 [M + NH_4_]^+^, 650.8 [M + Na]^+^.

For the detailed characterization of mass spectra, mass-mass spectra, ^1^H-NMR and HPLC of Analog-2, refer to Figures S2B, S3B, S4B and S5B.

#### 4.1.27. Synthesis of Cyclo(Me-Leu-Leu-Phe-Me-d-Leu*-*Leu) (Analog-3)

The experimental procedure was followed as described in the synthesis of Analog-1. In this synthesis, P1 was used instead of P5. Finally, 17.0 mg white powder with a yield of 38.5% were obtained. ^1^H-NMR (300 MHz, CDCl_3_), *δ*_H_ 7.97 (d, *J* = 9.1 Hz, 1H), 7.73 (dd, *J* = 5.7, 3.3 Hz, 1H), 7.55 (dd, *J* = 5.7, 3.3 Hz, 1H), 7.25 (dd, *J* = 7.6, 3.4 Hz, 4H), 6.20 (d, *J* = 9.4 Hz, 1H), 4.24 (dd, *J* = 5.9, 3.7 Hz, 1H), 3.59 (dt, *J* = 9.6, 6.7 Hz, 1H), 3.26 (dd, *J* = 8.1, 5.2 Hz, 1H), 3.10 (s, 2H), 2.96 (s, 2H), 1.59–1.40 (m, 8H), 1.32 (dd, *J* = 19.1, 8.3 Hz, 6H), 0.94 (dd, *J* = 8.7, 4.4 Hz, 20H), 0.83 (dd, *J* = 8.1, 6.7 Hz, 6H); ^13^C-NMR (75 MHz, CDCl_3_), *δ*_C_ 172.9, 172.8, 171.3, 171.1, 168.8, 136.1, 130.9, 129.5, 128.8 (2C), 128.6 (2C), 127.0, 68.2, 64.0, 59.0, 55.1, 49.7, 47.3, 41.7, 39.1, 38.7, 37.1, 30.6, 30.4, 28.9, 25.3, 25.0, 24.8, 23.8, 23.3, 23.0, 22.9, 22.7, 22.6, 22.5, 21.8; MS (ESI) *m*/*z*: 628.8 [M + H]^+^, 645.8 [M + NH_4_]^+^, 650.8 [M + Na]^+^.

For the detailed characterization of mass spectra, mass-mass spectra, ^1^H-NMR and HPLC of Analog-3, refer to Figures S2C, S3C, S4C and S5C.

#### 4.1.28. Synthesis of Cyclo(Me*-*Leu-Leu-Phe-Me-Leu-d*-*Leu) (Analog-4)

The experimental procedure was followed as described in the synthesis of Analog-1. In this synthesis, P2 was used instead of P5. Finally, 17.6 mg white powder with a yield of 34.2% were obtained. ^1^H-NMR (300 MHz, CDCl_3_), *δ*_H_ 7.28–7.17 (m, 5H), 6.95 (d, *J* = 9.8 Hz, 1H), 6.69 (d, *J* = 9.9 Hz, 1H), 5.28 (dd, *J* = 17.0, 8.1 Hz, 1H), 5.12 (t, *J* = 7.7 Hz, 1H), 4.90 (dd, *J* = 16.4, 8.6 Hz, 1H), 4.76 (dd, *J* = 15.4, 7.1 Hz, 1H), 4.59 (dd, *J* = 8.7, 6.1 Hz, 1H), 4.37 (dd, *J* = 17.4, 7.7 Hz, 1H), 3.22 (dd, *J* = 13.1, 8.3 Hz, 1H), 3.05–2.96 (m, 1H), 2.81 (s, 3H), 2.61 (s, 3H), 1.66–1.41 (m, 8H), 1.39–1.21 (m, 4H), 1.00–0.79 (m, 24H); ^13^C-NMR (75 MHz, CDCl_3_), *δ*_C_ 173.4, 172.1, 170.6, 169.6, 167.8, 136.2, 129.2 (2C), 128.8, 128.7, 127.1, 58.5, 53.8, 51.7, 49.2, 48.4, 40.9, 40.6, 38.7, 38.0, 34.2, 29.1 (2C), 25.0, 24.9, 24.8, 24.7, 24.6, 23.0, 22.9, 22.7, 22.6, 22.5, 22.2, 22.1; MS (ESI) *m*/*z*: 628.8 [M + H]^+^, 645.8 [M + NH_4_]^+^, 650.8 [M + Na]^+^.

For the detailed characterization of mass spectra, mass-mass spectra, ^1^H-NMR and HPLC of Analog-4, refer to Figures S2D, S3D, S4D and S5D.

#### 4.1.29. Synthesis of Cyclo(Me*-*d*-*Leu-Leu-Phe-Me-Leu-Leu (Analog-5)

The experimental procedure was followed as described in the synthesis of Analog-1. In this synthesis, P3 was used instead of P5. Finally, 39 mg white powder with a yield of 36.5% were obtained. ^1^H-NMR (300 MHz, CDCl_3_), *δ*_H_ 7.66 (d, *J* = 8.7 Hz, 1H), 7.28–7.17 (m, 5H), 6.74 (d, *J* = 9.8 Hz, 1H), 5.26–5.15 (m, 1H), 5.11 (dd, *J* = 9.4, 6.2 Hz, 1H), 5.00 (td, *J* = 8.4, 6.4 Hz, 1H), 4.85–4.79 (m, 1H), 4.63–4.50 (m, 1H), 4.46 (dd, *J* = 10.4, 6.6 Hz, 1H), 3.30–3.17 (m, 1H), 2.96 (t, *J* = 5.7 Hz, 1H), 2.84 (s, 3H), 2.54 (s, 3H), 2.12–1.76 (m, 4H), 1.62–1.48 (m, 8H), 1.01–0.75 (m, 24H); ^13^C-NMR (75 MHz, CDCl_3_), *δ*_C_ 173.5, 171.8, 171.5, 169.6, 167.9, 137.3, 129.6, 129.4, 128.3 (2C), 126.5, 58.3, 53.8, 51.7, 51.1, 45.8, 41.0, 38.6, 37.8, 37.2, 34.0, 29.7, 29.1, 25.4 24.9, 24.8, 24.7, 24.4, 23.3, 23.0, 22.9, 22.6, 22.4, 22.2, 21.9; MS (ESI) *m*/*z*: 628.8 [M + H]^+^, 645.8 [M + NH_4_]^+^, 650.8 [M + Na]^+^.

For the detailed characterization of mass spectra, mass-mass spectra, ^1^H-NMR and HPLC of Analog-5, refer to Figures S2E, S3E, S4E and S5E.

#### 4.1.30. Synthesis of Cyclo(Me*-*Leu-d-Leu-Phe-Me-Leu-Leu) (Analog-6)

The experimental procedure was followed as described in the synthesis of Analog-1. In this synthesis,P4 was used instead of P5. Yield: 42.5%. ^1^H-NMR (300 MHz, CDCl_3_), *δ*_H_ 7.28–7.17 (m, 5H), 7.03–6.87 (m, 1H), 6.71 (d, *J* = 9.6 Hz, 1H), 5.25 (dd, *J* = 16.3, 8.2 Hz, 1H), 5.11 (t, *J* = 7.6 Hz, 1H), 4.91 (t, *J* = 7.6 Hz, 1H), 4.76 (dd, *J* = 15.5, 7.0 Hz, 1H), 4.57 (dd, *J* = 8.8, 5.9 Hz, 1H), 4.34 (dd, *J* = 17.1, 7.6 Hz, 1H), 3.21 (dd, *J* = 13.0, 8.6 Hz, 2H), 2.79 (s, 3H), 2.60 (s, 3H), 1.53 (ddd, *J* = 17.6, 12.8, 7.0 Hz, 8H), 1.42–1.22 (m, 4H), 1.00–0.79 (m, 24H); ^13^C-NMR (75 MHz, CDCl_3_), *δ*_C_ 173.3, 172.0, 170.9, 169.9, 167.7, 136.1, 129.2 (2C), 128.8 (2C), 127.2, 58.5, 53.8, 51.8, 50.2, 49.3, 48.4, 40.9, 40.4, 37.9, 34.2, 29.1, 25.1, 24.9, 24.8, 24.6, 23.0, 22.9, 22.7, 22.6, 22.4, 22.4, 22.2, 22.1, 21.7; MS (ESI) *m*/*z*: 628.8 [M + H]^+^, 645.8 [M + NH_4_]^+^, 650.8 [M + Na]^+^.

For the detailed characterization of mass spectra, mass-mass spectra, ^1^H-NMR and HPLC of Analog-6, refer to Figures S2F, S3F, S4F and S5F.

### 4.2. In Vitro Cell Experiments

#### 4.2.1. Cell Culture

Human hepatocellular cells (HepG_2_), human breast cancer cells (MCF-7), human breast adenocarcinoma cells (MDA-MB-435) and a human cervical carcinoma cell line (Hela) were selected as our in vitro cancerous cell models. The cells were all cultured in DMEM medium containing 10% fetal bovine serum, 1% penicillin-streptomycin and antifungal agent, incubated at 37 °C with 5% CO_2_ and 90% humidity.

#### 4.2.2. MTT Measurement

The cytotoxicity of Galaxamide and its analogs on the four cancerous cells was evaluated using the MTT method. Briefly, cells were seeded in 96-well tissue culture plates at a concentration of 2.5 × 10^3^ cells/well. When cells’ confluence reached 70%, Galaxamide and its analogs with a concentration of 0, 5, 10 and 15 µg/mL were added to the cell cultures and incubated for 48 h. After that, 20 µL MTT reagents were added to each well and incubated between 3–5 h according to the nature of cells; the color intensity of the cell culture at 570 nm was measured using a microplate spectrophotometer. Finally, the cell viability was calculated accordingly. Note: MTT is 3-(4,5-dimethyl-2-thiazolyl)-2,5-diphenyl-2-*H*-tetrazolium bromide.

#### 4.2.3. Flow Cytometry Experiments

HepG_2_ cells were seeded in 6-well tissue culture plates at a concentration of 1 × 10^5^ cells/well. Galaxamide and its analogs with a concentration of 0, 5, 10 and 15 µg/mL were added to the cell cultures and incubated for 48 h. After that, 5 µL Annexin V-fluorescein isothiocyanate (FITC) and 5 µL of PI were added for dual staining of the cells and analyzed by FACScan flow cytometry. Cell cycle and apoptotic cells were determined by counting the FITC-positive and PI-negative fractions.

#### 4.2.4. Hoechst 33342 Staining

HepG_2_ cells were seeded in 6-well tissue culture plates at a concentration of 1 × 10^5^ cells/well. Galaxamide and its analogs with a concentration of 0, 5, 10 and 15 µg/mL were added to the cell cultures and incubated for 48 h. After that, cells were fixed with 3.7% of paraformaldehyde for 10 min and washed three times with PBS. Then, Hoechst 33342 was added for cell staining, then visualized using a fluorescent microscope.

#### 4.2.5. Western Blot Experiments

Target proteins were first extracted using Radio-Immunoprecipitation Assay (RIPA) buffer and quantified by the Bradford method. Then, proteins were transferred to a polyvinylidene difluoride (PVDF) membrane, and the membrane was blocked with 5% non-fat milk for 1 h and incubated with a primary antibody (Cell Signaling Technology, Danvers, MA, USA, rabbit monoclonal antibody, 1:1000) at 4 °C overnight. After that, the membrane was washed two times with TBST buffer (Tris-buffered saline with 0.1% Tween-20) and incubated with a secondary antibody (goat-anti-rabbit horseradish peroxidase (HRP)-conjugated 1:15,000) for 1 h under room temperature. Finally, the membrane was washed three times with TBST, and the intensity of the specific immunoreactive bands was detected by enhanced chemiluminescence.

#### 4.2.6. Statistical Analysis

All data in the manuscript were expressed in the form of the mean ± the standard deviation. The statistical analysis were performed using Student’s *t*-test, and differences between groups were considered significant when *p* < 0.05.

## 5. Conclusions

In the present work, we synthesized six Galaxamide analogs using a classic “3 + 2” synthetic strategy, where five of the analogs were designed with the varied position of the d-amino acid. Their anticancer activity was tested against four human cancer cell lines, such as HepG_2_, MCF-7, MDA-MB-435 and Hela cells. We found that changes in the d-amino acid position resulted in the alteration of the anticancer potential. Analog-2 (with a d-phenylalanine at position 1), Analog-4 (with a d-leucine at position 3) and Analog-6 (with a d-leucine at position 5) showed improved anticancer activity compared to natural product Galaxamide. Among those analogs, Analog-6 exhibited the best anticancer effects. Analog-6 could cause the early apoptosis of HepG_2_ cells by inhibiting their growth in the sub-G1 stage of the cell cycle and induced chromatin condensation. Moreover, Analog-6 exerted the early apoptotic effect through a mitochondria-mediated pathway. The findings in our work will provide a database for the rational design of Galaxamide analogs with improved anticancer activity that are promising for the development of anticancer drugs.

## Figures and Tables

**Table 1:** Cytotoxicity of Galaxamide and its analogs on different human cancer cell lines.

Compound	IC_50_ (µg/mL) in Different Cell Lines			
	HepG_2_	MCF-7	Hela	MDA-MB-435
Galaxamide	9.45 ± 0.25	14.08 ± 0.28	4.94 ± 0.19	11.08 ± 0.22
Analog-1	5.85 ± 0.13	13.03 ± 0.33	6.85 ± 0.26	9.76 ± 0.18
Analog-2	4.56 ± 0.22	9.64 ± 0.27	4.95 ± 0.18	9.5 ± 0.32
Analog-3	7.71 ± 0.11	18.16 ± 0.33	4.39 ± 0.26	12.6 ± 0.29
Analog-4	7.2 ± 0.15	8.62 ± 0.26	4.2 ± 0.32	7.67 ± 0.18
Analog-5	10.72 ± 0.32	15.21 ± 0.29	7.4 ± 0.33	20.43 ± 0.23
Analog-6	4.1 ± 0.20	8.15 ± 0.18	3.42 ± 0.25	6.76 ± 0.22

## Supplementary Materials

Supplementary materials can be found at www.mdpi.com/1422-0067/18/3/544/s1.
