# Supplementary material for: d-Amino Acid Position Influences the Anticancer Activity of Galaxamide Analogs: An Apoptotic Mechanism Study
Source: Int J Mol Sci. 2017 Mar 10;18(3):544. doi: 10.3390/ijms18030544 (PMC5372560; doi:10.3390/ijms18030544)

Supporting Information

D-amino acid position influences the anticancer activity of *Galaxamide* analogs: An apoptotic mechanism study

Defa Bai, Siming Yu, Shenghui Zhong, Bingxin Zhao,
Shaoling Qiu, Jianwei Chen, Jignesh Lunagariya, Xiaojian Liao,
Shihai Xu

Figure S1. Quantification and statistical analysis of Hoechst data for comparing the percentage of cells with regular nuclei shape treated by *Galaxamide* and Analog-6,
* $p < 0.5$, ** $p < 0.01$.

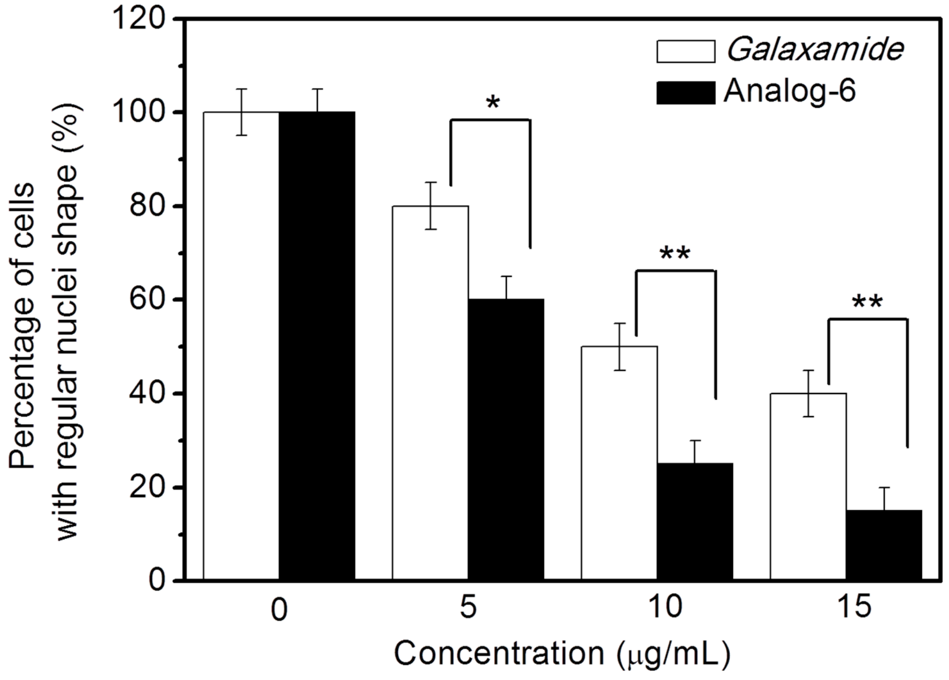

Figure S2. Mass Spectra (MS) of Analog-1 to Analog-6 (A-F)

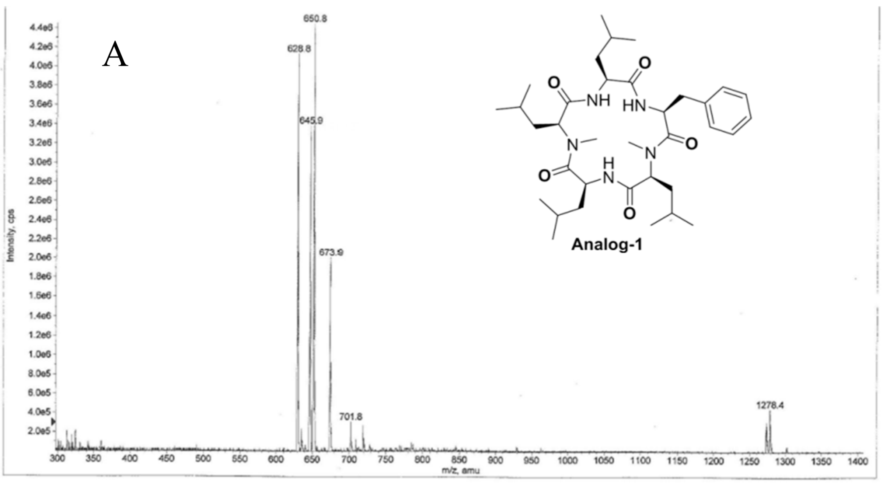

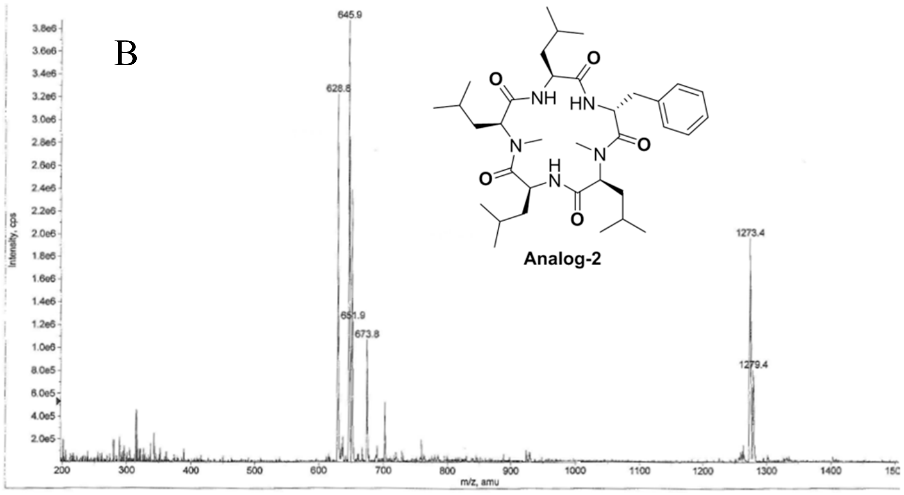

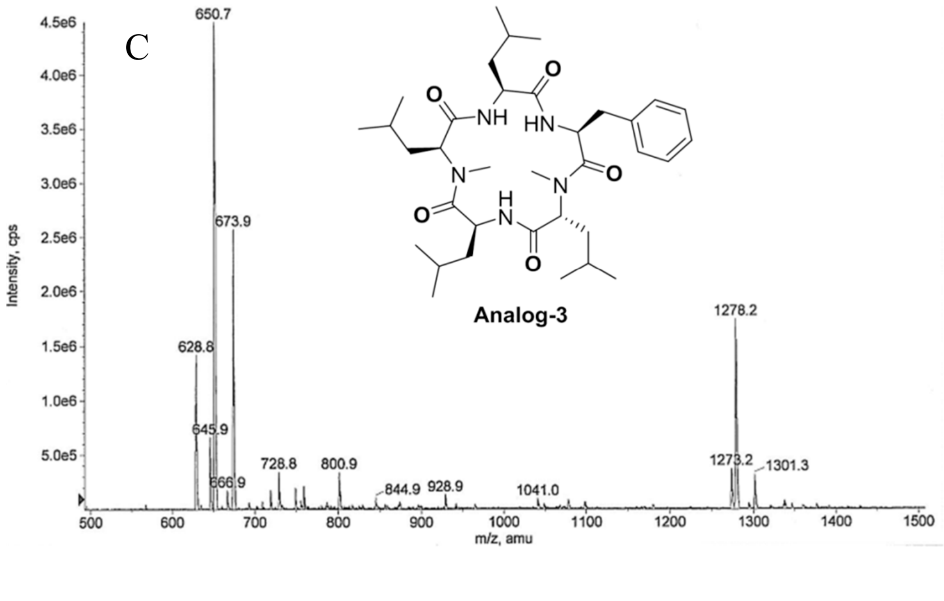

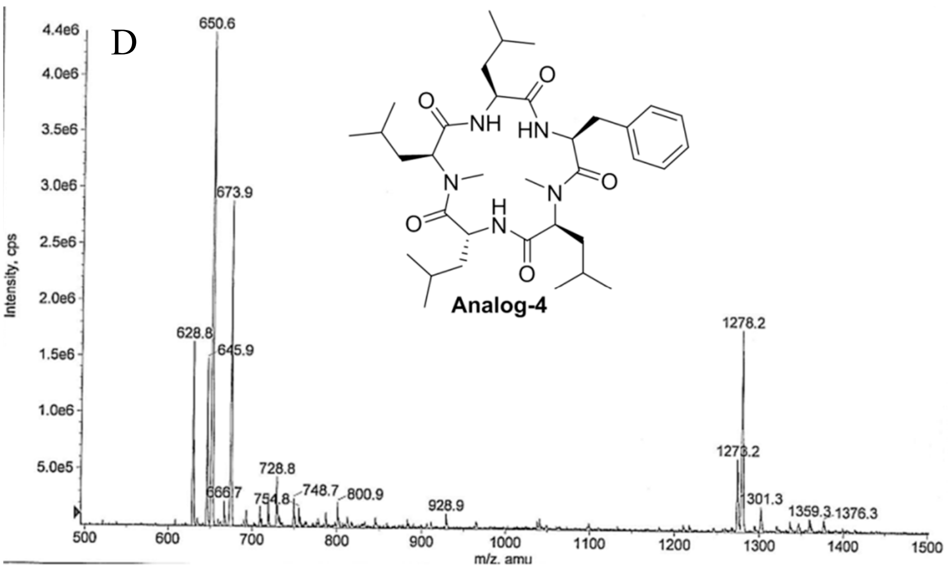
D
Analog-4
Intensity, cps
m/z, amu
650.6
673.9
628.8
645.9
1278.2
1273.2
728.8
666.7
754.8
748.7
800.9
928.9
1301.3
1359.3
1376.3

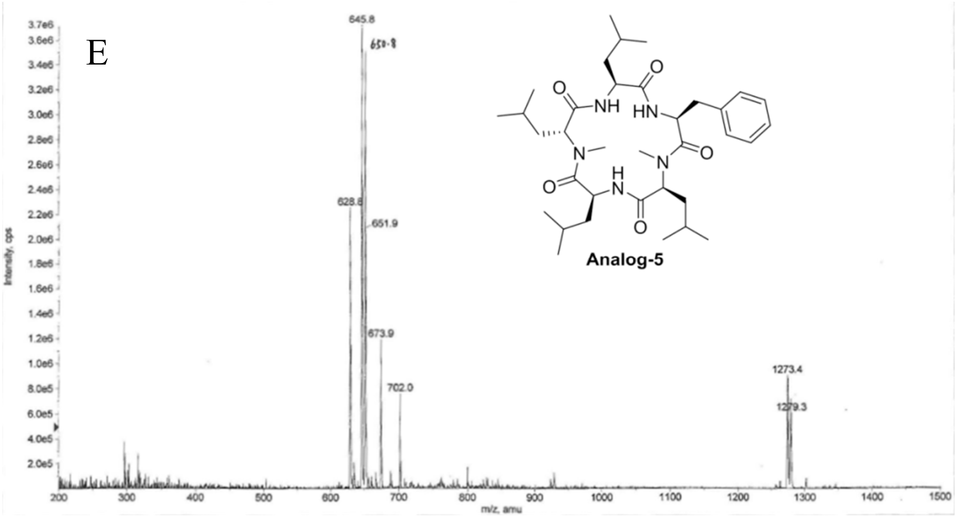
E
Analog-5
645.8
628.8
651.9
673.9
702.0
1273.4
1279.3

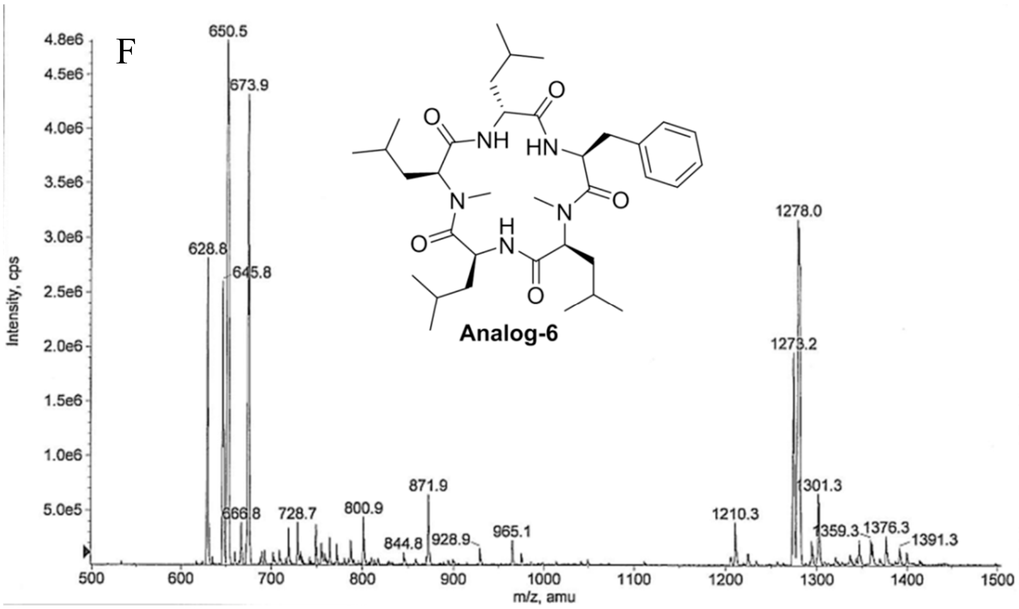
F
Analog-6
Intensity, cps
m/z, amu
650.5
673.9
628.8
645.8
1278.0
1273.2
1301.3
666.8
728.7
800.9
871.9
844.8
928.9
965.1
1210.3
1359.3
1376.3
1391.3

Figure S3. Mass-Mass Spectra (MS-MS) of Analog-1 to Analog-6 (A-F)

A

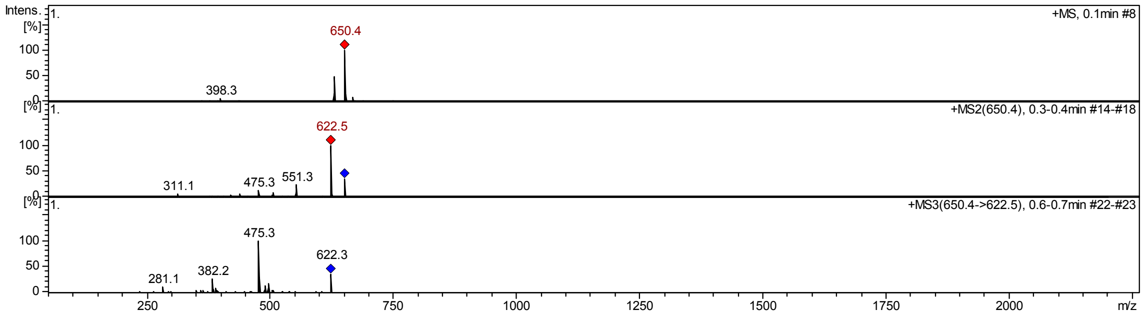

B

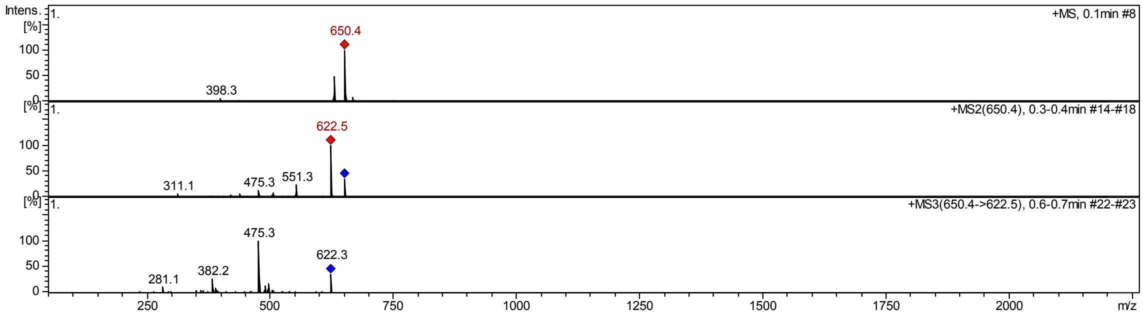

C

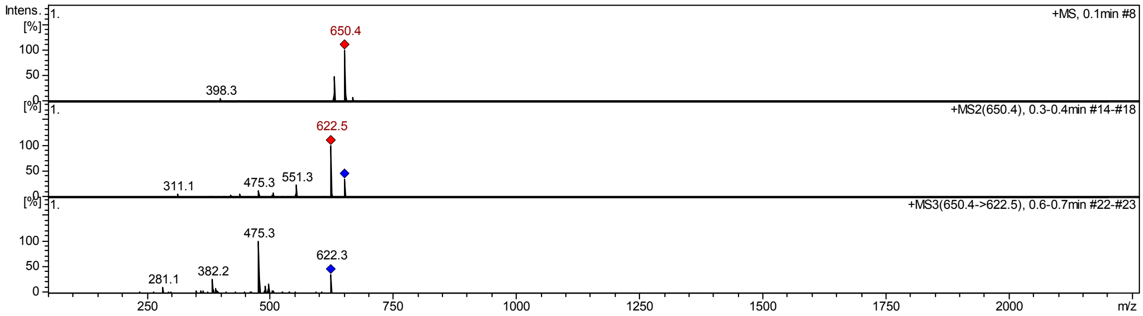

D

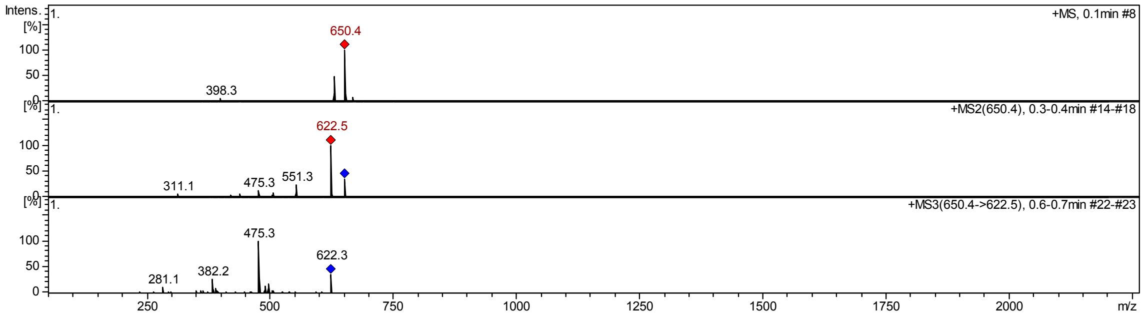

E

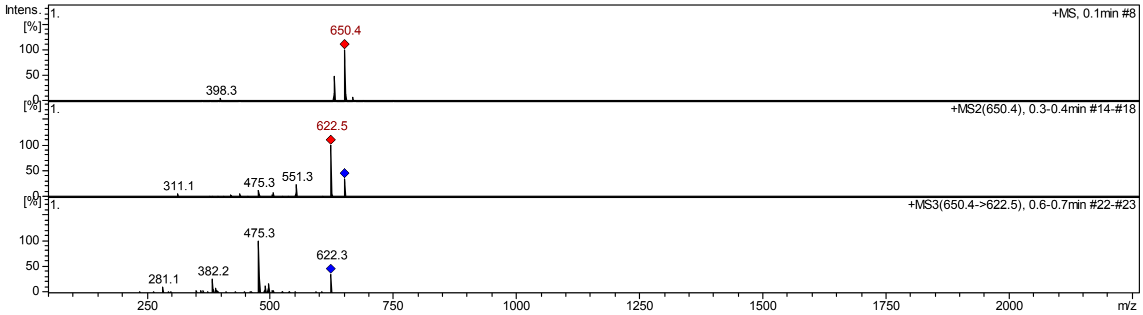

F

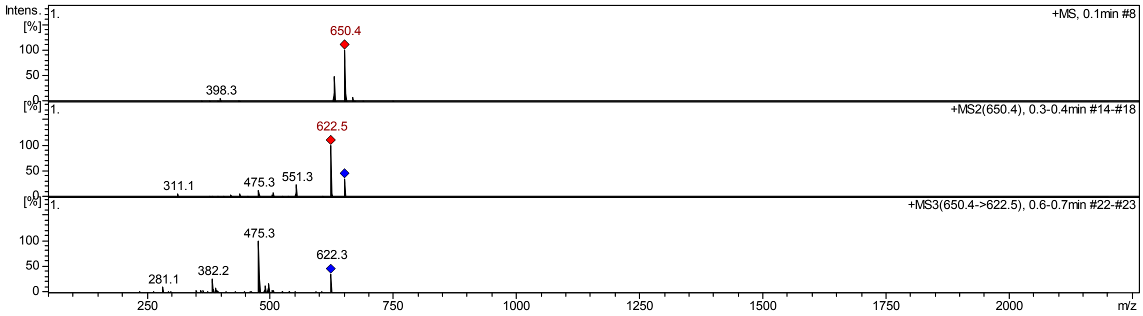

Figure S4. ^{1}H-NMR of Analog-1 to Analog-6 (A-F)

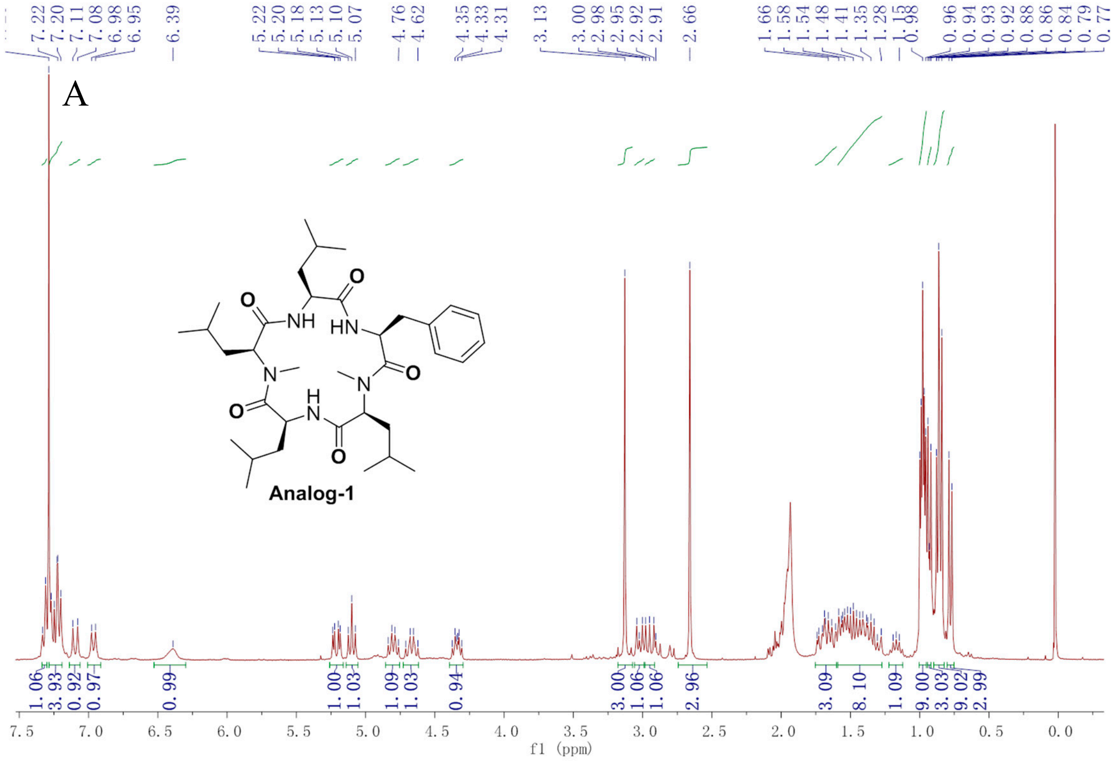

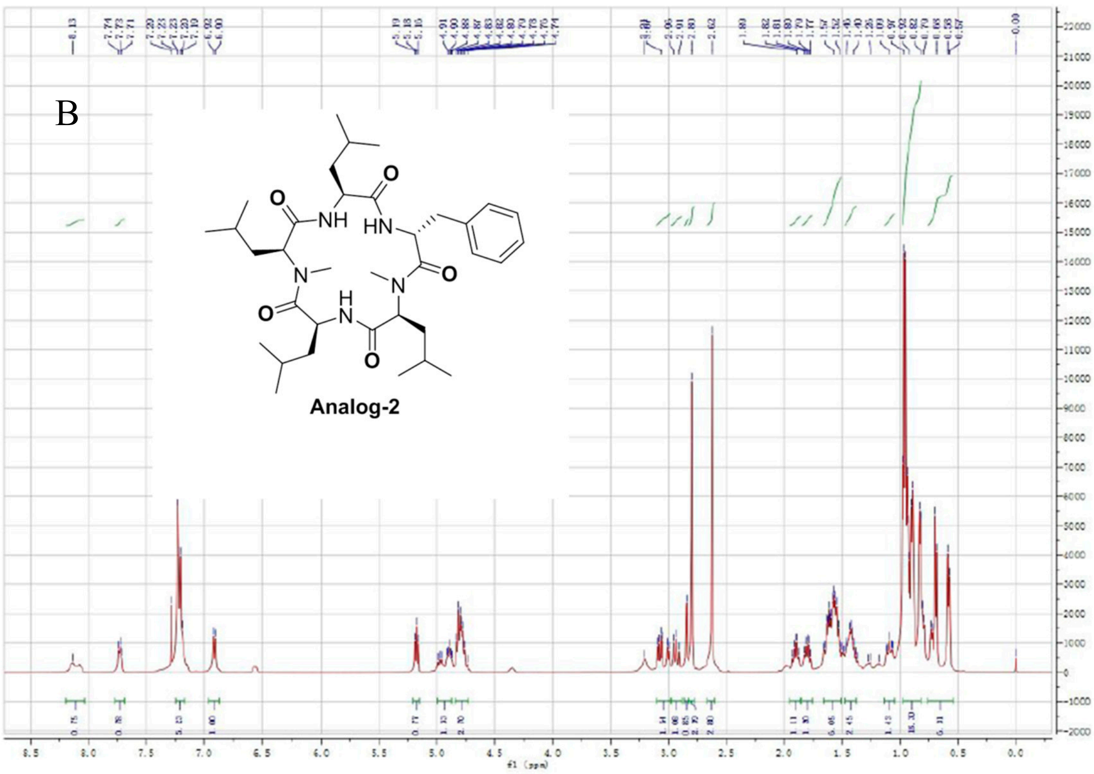
B
Analog-2
f1 (ppm)

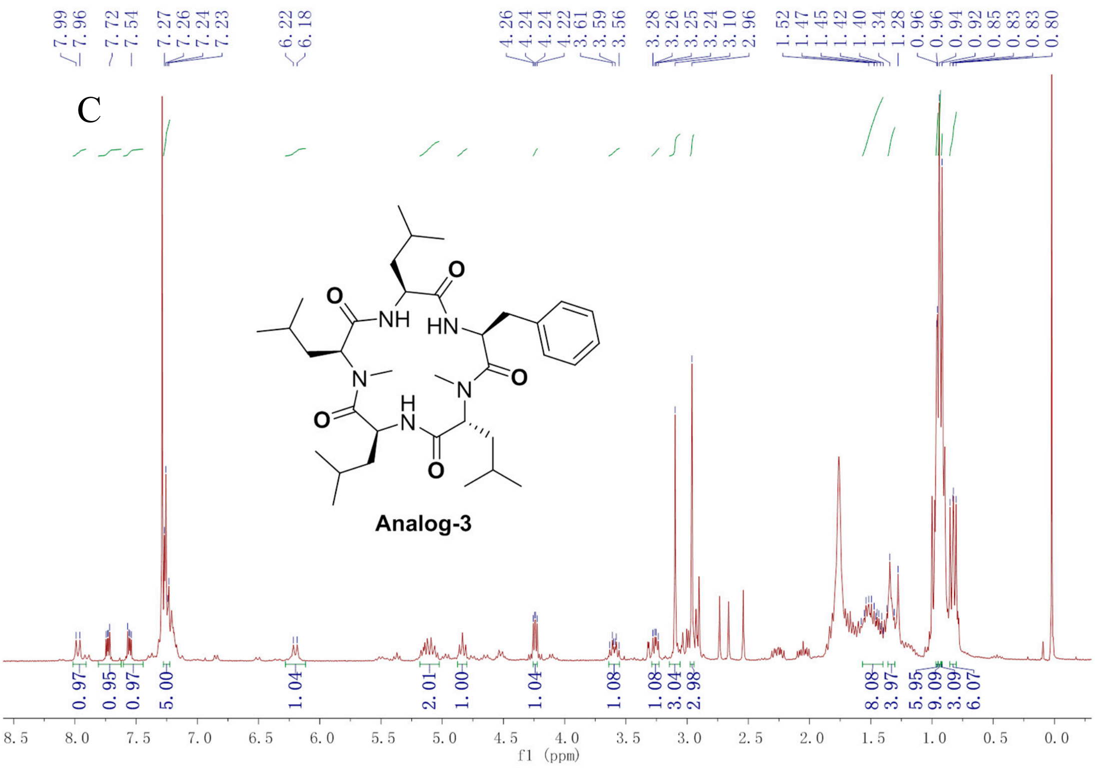
C
Analog-3
f1 (ppm)

D

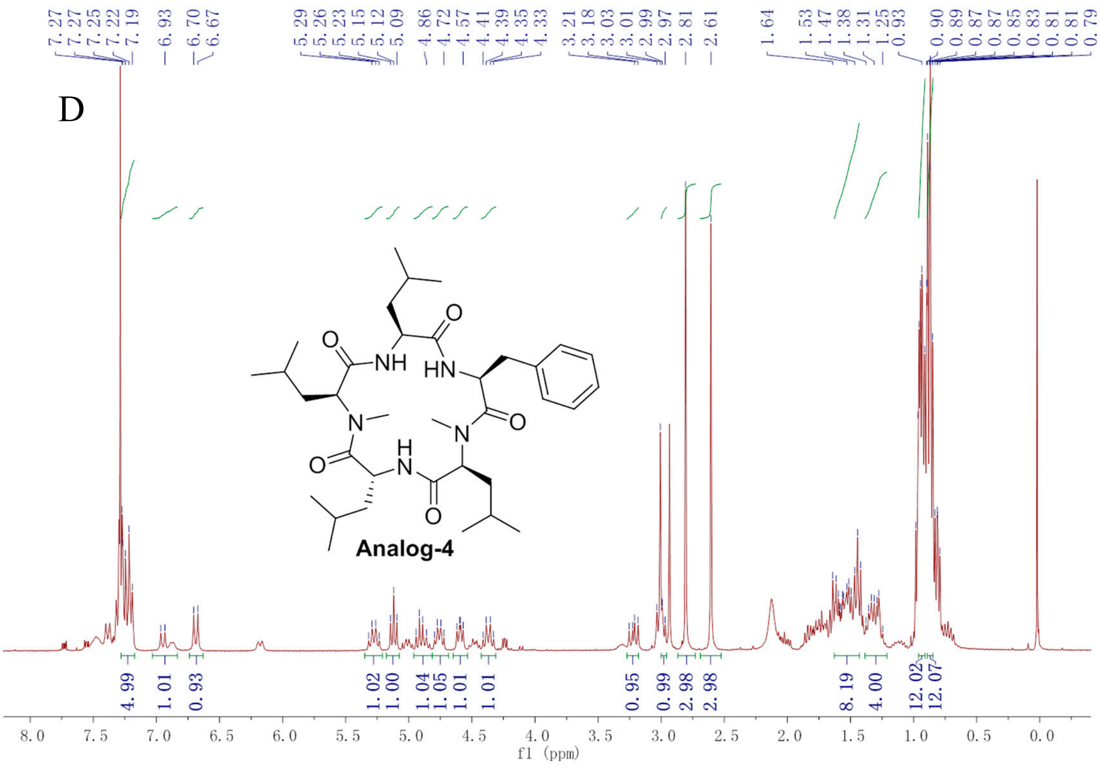
7.27 7.27 7.25 7.22 7.19 6.93 6.70 6.67 5.29 5.26 5.23 5.15 5.12 5.09 4.86 4.72 4.57 4.41 4.39 4.35 4.33 3.21 3.18 3.03 3.01 2.99 2.97 2.81 2.61 1.64 1.53 1.47 1.38 1.31 1.25 0.93 0.90 0.89 0.87 0.85 0.83 0.81 0.81 0.79
NH
HN
N
O
Analog-4
4.99 1.01 0.93 1.02 1.00 1.04 1.05 1.01 1.01 0.95 0.99 2.98 2.98 8.19 4.00 12.02 12.07
8.0 7.5 7.0 6.5 6.0 5.5 5.0 4.5 4.0 3.5 3.0 2.5 2.0 1.5 1.0 0.5 0.0
f1 (ppm)

E

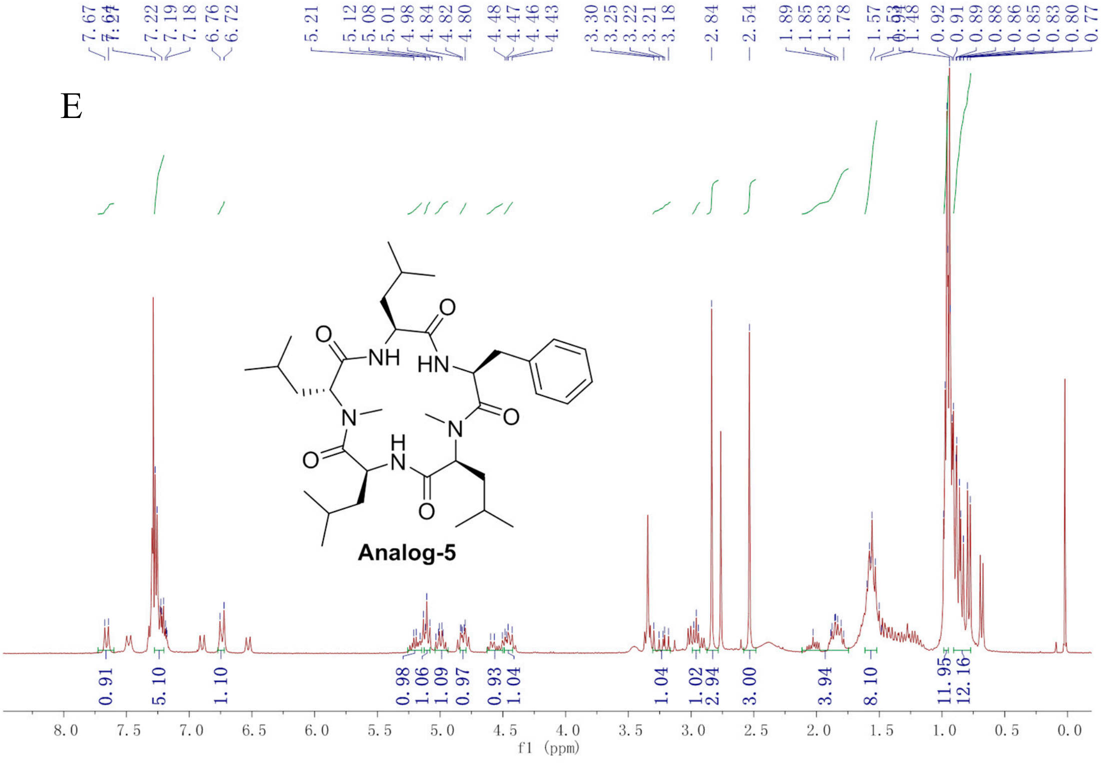
7.67 7.64 7.27 7.22 7.19 7.18 6.76 6.72 5.21 5.12 5.08 5.01 4.98 4.84 4.82 4.80 4.48 4.47 4.46 4.43 3.30 3.25 3.22 3.21 3.18 2.84 2.54 1.89 1.85 1.83 1.78 1.57 1.48 0.92 0.91 0.89 0.88 0.86 0.85 0.83 0.80 0.77
NH
HN
N
O
Analog-5
0.91 5.10 1.10 0.98 1.06 1.09 0.97 0.93 1.04 1.04 1.02 2.94 3.00 3.94 8.10 11.95 12.16
8.0 7.5 7.0 6.5 6.0 5.5 5.0 4.5 4.0 3.5 3.0 2.5 2.0 1.5 1.0 0.5 0.0
f1 (ppm)

F

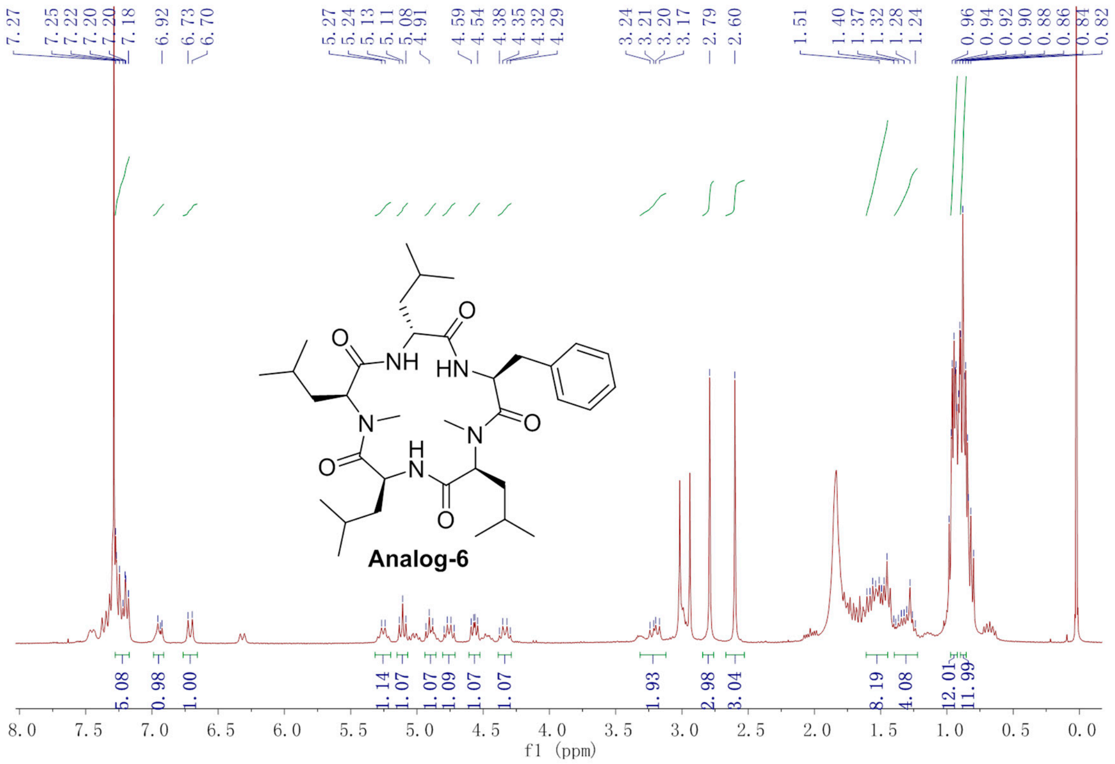

Figure S5. HPLC of Analog-1 to Analog-6 (A-F)

Note: HPLC conditions: Mobile phase flow rate：2 mL/min, Injection volume：20 Ml, Equipment：Agilent 1200 serious HPLC, Column：5C_{18}-MS-II (4.61ID × 250 mm)

A

峰 #	保留时间 [min]	类型	峰宽 [min]	峰面积 [mAU*s]	峰高 [mAU]	峰面积 %
1	4.454	BB	0.1224	9.75937	1.09026	0.7733
2	5.121	BB	0.1370	20.68487	2.29889	1.6390
3	6.436	BB	0.2458	55.94862	3.29945	4.4331
4	7.256	BB	0.1138	8.46250	1.15020	0.6705
5	17.072	BB	0.3160	1167.21814	57.22709	92.4842
总量 ：				1262.07350	65.06589	

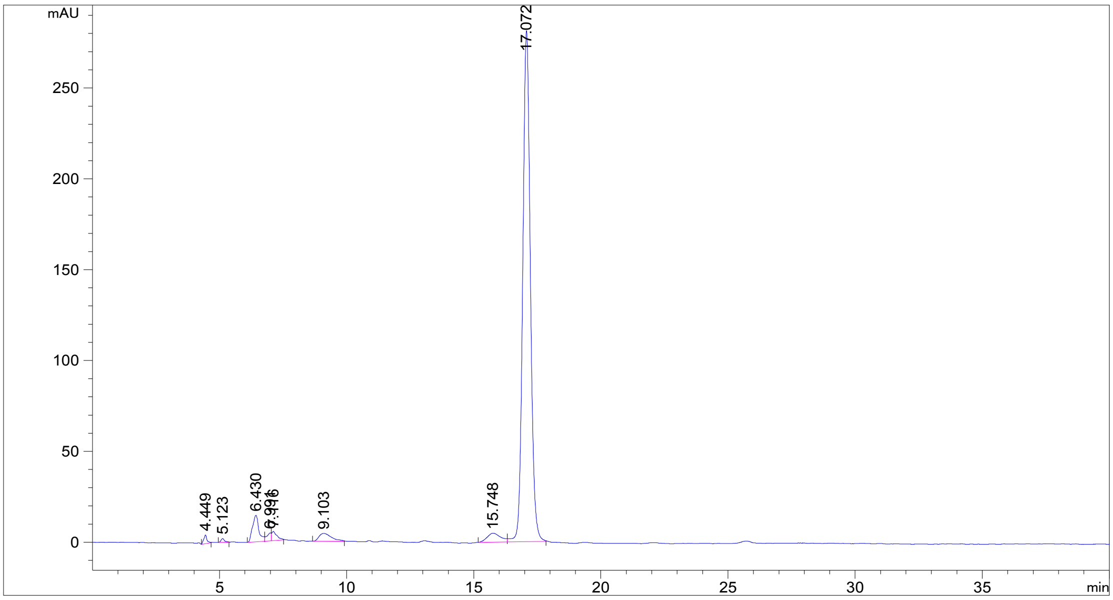

B

峰 #	保留时间 [min]	类型	峰宽 [min]	峰面积 [mAU*s]	峰高 [mAU]	峰面积 %
1	4.348	BB	0.1178	9.34541	1.09241	0.2902
2	6.422	BB	0.2361	46.14788	2.92418	1.4329
3	17.108	BB	0.3179	3089.35693	150.23499	95.9273
4	23.280	BV	0.2506	29.10389	1.56732	0.9037
5	23.754	VV	0.2087	22.13979	1.35448	0.6875
6	24.214	VB	0.1577	14.07874	1.17551	0.4372
7	24.735	BB	0.1427	10.34518	1.01672	0.3212

总量 : 3220.51782 159.36560

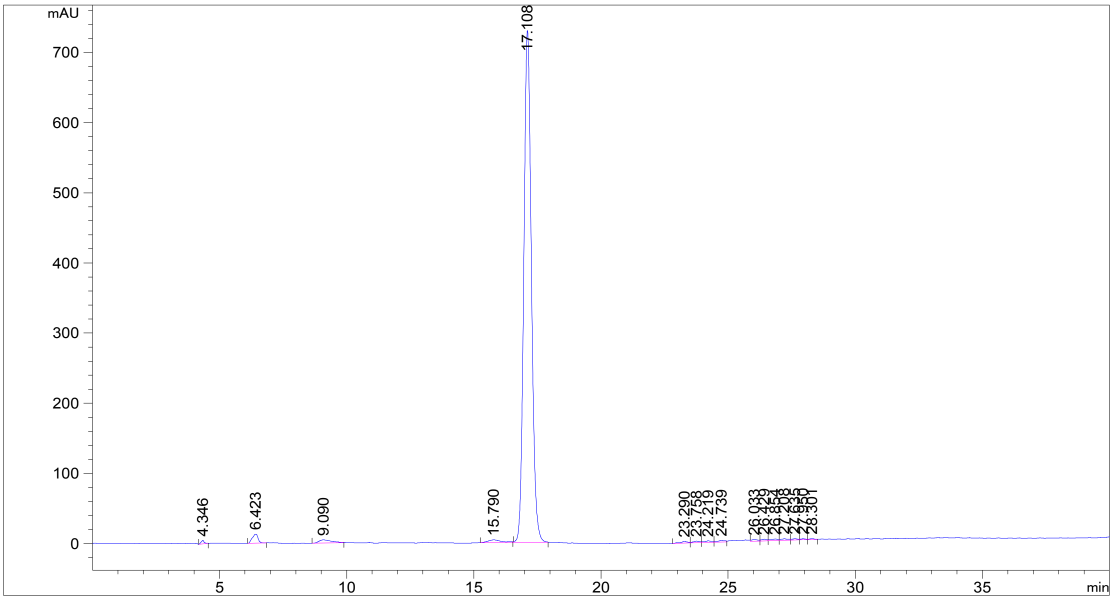

C

峰 #	保留时间 [min]	类型	峰宽 [min]	峰面积 [mAU*s]	峰高 [mAU]	峰面积 %
1	4.376	BB	0.1276	10.11250	1.09680	0.3178
2	6.388	BB	0.2163	81.75143	5.28897	2.5690
3	16.642	BB	0.3095	3090.30908	154.37335	97.1132

总量 : 3182.17301 160.75911

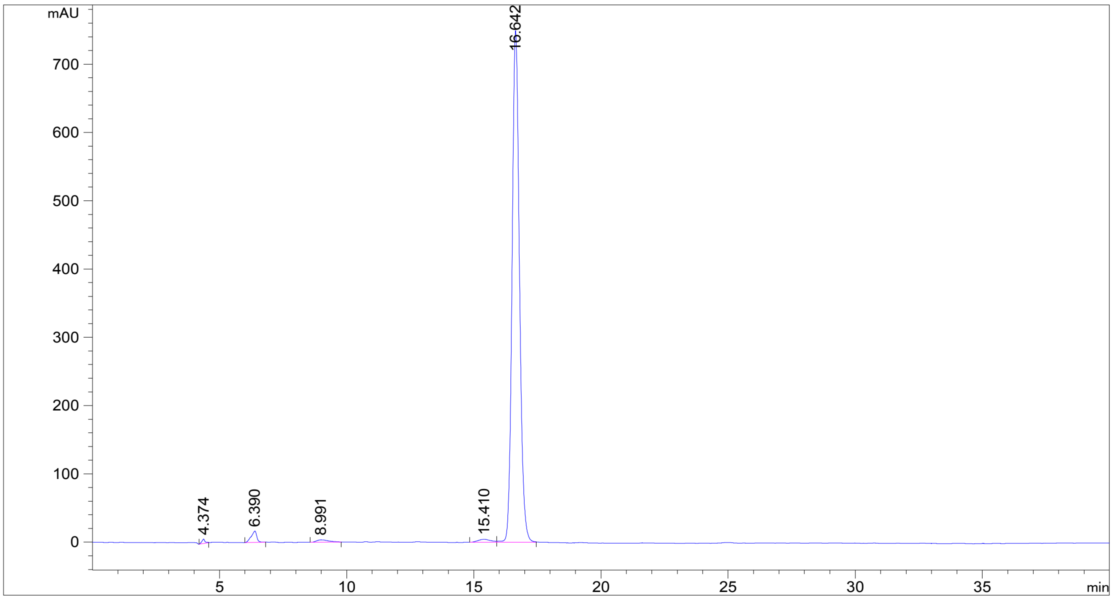

D

峰 #	保留时间 [min]	类型	峰宽 [min]	峰面积 [mAU*s]	峰高 [mAU]	峰面积 %
1	4.356	BB	0.1283	11.49923	1.23823	0.3295
2	6.388	BB	0.2216	82.26740	5.17070	2.3574
3	16.634	BB	0.3139	3396.03931	167.94440	97.3131

总量 : 3489.80593 174.35333

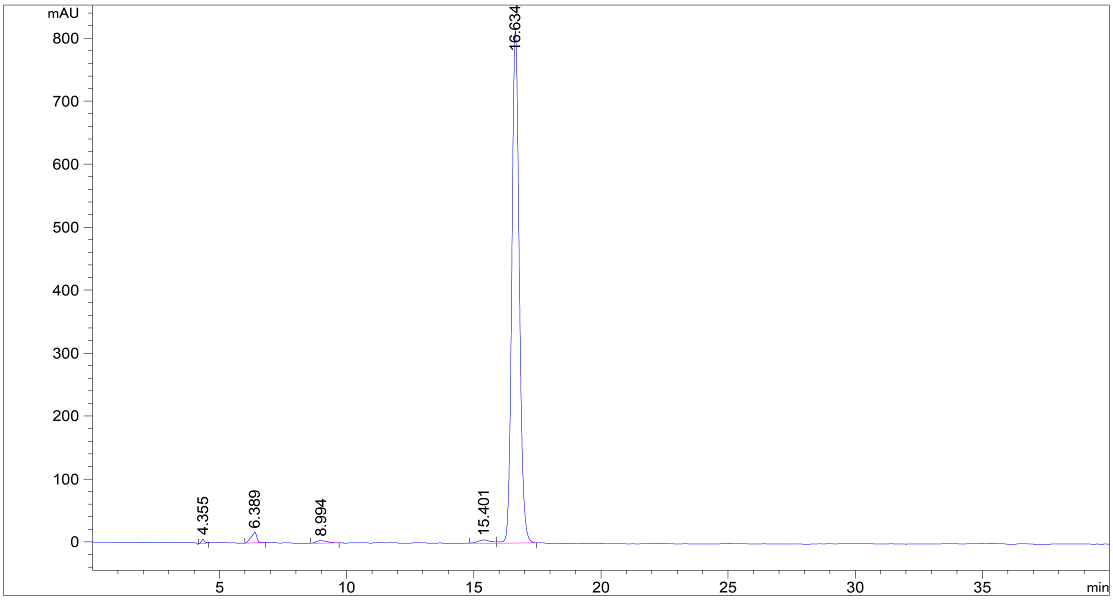

E 峰 #	保留时间 [min]	类型	峰宽 [min]	峰面积 [mAU*s]	峰高 [mAU]	峰面积 %
1	4.345	BB	0.1379	13.36476	1.32300	0.3517
2	6.387	BB	0.2289	84.58970	5.11692	2.2258
3	16.648	BB	0.3172	3702.45483	180.61356	97.4225

总量 : 3800.40930 187.05348

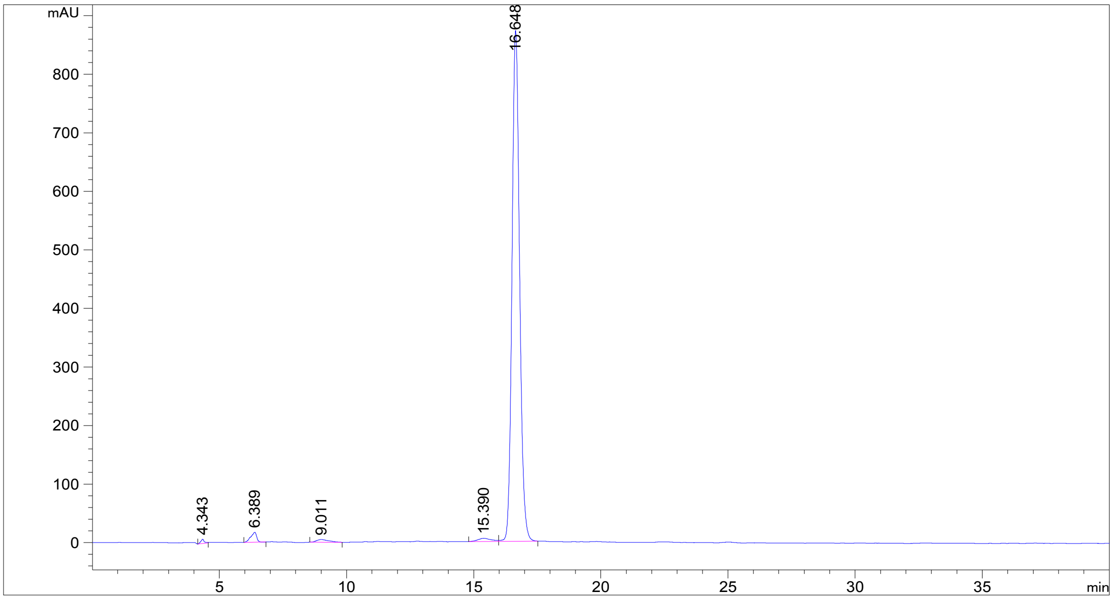

F

峰 #	保留时间 [min]	类型	峰宽 [min]	峰面积 [mAU*s]	峰高 [mAU]	峰面积 %
1	6.389	BB	0.2125	64.68988	4.32548	1.7245
2	16.666	BB	0.3100	3686.52856	183.77298	98.2755

总量 : 3751.21844 188.09846

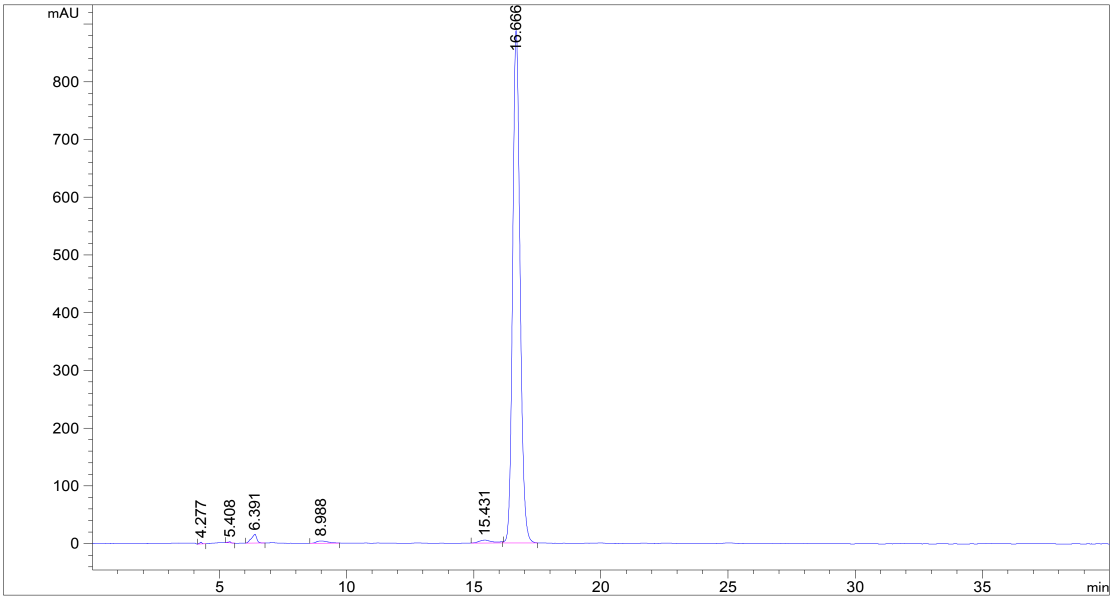

Supplement: Supplementary file 1 [file ijms-18-00544-s001.pdf]
